# Lethal perturbation of an *Escherichia coli* regulatory network is triggered by a restriction-modification system's regulator and can be mitigated by excision of the cryptic prophage Rac

**DOI:** 10.1093/nar/gkad1234

**Published:** 2023-12-28

**Authors:** Katarzyna Gucwa, Ewa Wons, Aleksandra Wisniewska, Marcin Jakalski, Zuzanna Dubiak, Lukasz Pawel Kozlowski, Iwona Mruk

**Affiliations:** Department of Microbiology, Faculty of Biology, University of Gdansk, Wita Stwosza 59, Gdansk 80-308, Poland; Department of Microbiology, Faculty of Biology, University of Gdansk, Wita Stwosza 59, Gdansk 80-308, Poland; Department of Microbiology, Faculty of Biology, University of Gdansk, Wita Stwosza 59, Gdansk 80-308, Poland; 3P-Medicine Laboratory, Medical University of Gdansk, Debinki 7, 80-211 Gdansk, Poland; Department of Microbiology, Faculty of Biology, University of Gdansk, Wita Stwosza 59, Gdansk 80-308, Poland; Institute of Informatics, Faculty of Mathematics, Informatics and Mechanics, University of Warsaw, Banacha 2, 02-097 Warsaw, Poland; Department of Microbiology, Faculty of Biology, University of Gdansk, Wita Stwosza 59, Gdansk 80-308, Poland

## Abstract

Bacterial gene regulatory networks orchestrate responses to environmental challenges. Horizontal gene transfer can bring in genes with regulatory potential, such as new transcription factors (TFs), and this can disrupt existing networks. Serious regulatory perturbations may even result in cell death. Here, we show the impact on *Escherichia coli* of importing a promiscuous TF that has adventitious transcriptional effects within the cryptic Rac prophage. A cascade of regulatory network perturbations occurred on a global level. The TF, a C regulatory protein, normally controls a Type II restriction-modification system, but in *E. coli* K-12 interferes with expression of the RacR repressor gene, resulting in de-repression of the normally-silent Rac *ydaT* gene. YdaT is a prophage-encoded TF with pleiotropic effects on *E. coli* physiology. In turn, YdaT alters expression of a variety of bacterial regulons normally controlled by the RcsA TF, resulting in deficient lipopolysaccharide biosynthesis and cell division. At the same time, insufficient RacR repressor results in Rac DNA excision, halting Rac gene expression due to loss of the replication-defective Rac prophage. Overall, Rac induction appears to counteract the lethal toxicity of YdaT. We show here that *E. coli* rewires its regulatory network, so as to minimize the adverse regulatory effects of the imported C TF. This complex set of interactions may reflect the ability of bacteria to protect themselves by having robust mechanisms to maintain their regulatory networks, and/or suggest that regulatory C proteins from mobile operons are under selection to manipulate their host's regulatory networks for their own benefit.

## Graphical Abstract

**Graphical Abstract:**
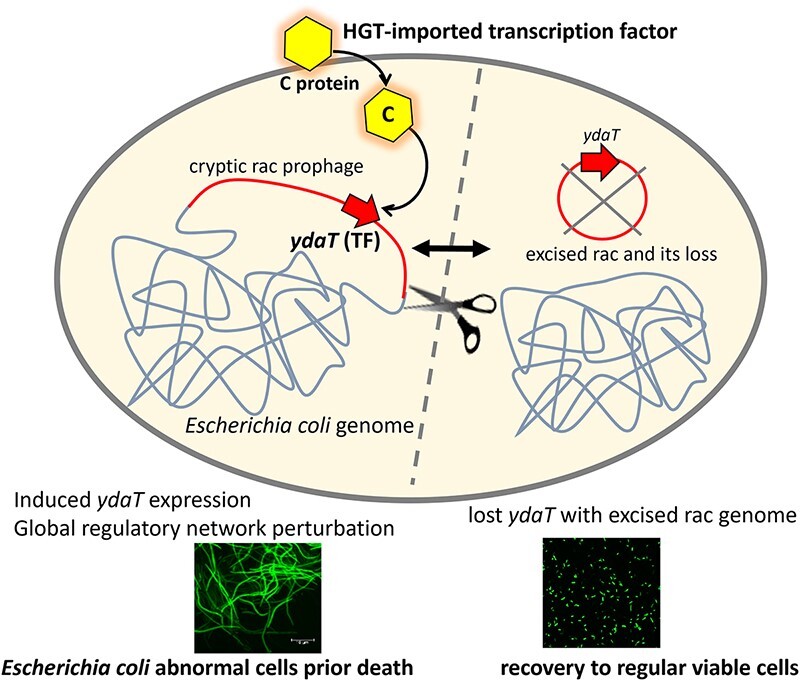

## Introduction

Prokaryotic genomes are highly plastic and can exploit horizontal gene transfer, point mutations, and gene rearrangements to adapt to environmental changes and other challenges. Being tolerant of constant gene influx is selected for, in order to preserve fitness in the absence of obligate sexual recombination (1). However, acquiring genetic modules that often include regulators, such as transcription factors (TFs), can pose challenges, as a new TF must not only exert its specific function, but also avoid disrupting the host's existing regulatory networks upon modification. Bacterial TFs collectively build up and coordinate flexible regulatory circuits (2,3). However, significant perturbations by a new TF of the existing regulatory orchestration and interconnectivity may result in reduced fitness and even cell death. Perhaps this is why regulatory networks are subject to continuous re-wiring and fine-tuning to optimize functionality (4). Still, we poorly understand the impact of exogenous TFs on horizontal gene transfer and gene regulatory network robustness, though mechanisms for temporary silencing of imported genes appears to be part of the general solution (5–7).

Another aspect of gene flux dynamics is associated with integration of bacteriophages into bacterial genomes, which then co-evolve with their hosts as prophages (8–11). Often prophage DNA remains as ‘foreign islands’ with other horizontally accepted gene fragments, resulting in host genomic mosaicism. Prophages often seem to be beneficial for the host, improving its survival, persistence, metabolic strategies and prevalence in microbial communities, yet still more needs to be explored (12,13). Genomic analysis of diverse bacterial species has revealed multiple prophages present within each genome in the form of ‘cryptic’ prophages, unable to proceed through a lytic cycle due to mutations accumulated during phage-host co-existence. The vast majority of such defects is related to process of phage DNA excision, virion assembly or host cell lysis (9). Still, even cryptic phage DNA undergoes positive genetic selection to maintain those of its genes that drive bacterial adaptation and fitness, whereas other genes are lost (10,14,15). Among characterized *E. coli* strains, *E. coli* O157:H7 str Sakai has the largest known number of prophages, harboring 18 that constitute almost 16% of the total genome (16). Well-studied *E. coli* K-12 has nine cryptic prophages, with DLP12, e14, Rac, CPZ-55, and Qin prophages as the best characterized (9,10).

In recent reports, we monitored the transfer of an operon into a new host, in real time to understand the fate of horizontally transferred DNA fragments. As a model, we used the Csp231I Type II restriction-modification (R-M) system (17). In this particular operon, the two enzymes comprising R-Ms—restriction endonuclease (REase) and DNA methyltransferase (MTase)—are precisely controlled at the transcriptional level by a dedicated TF, called a C protein (18), part of a large family of such proteins (19,20). The C protein not only activates and represses (depending on its concentration) the REase gene and its own as an autoregulator, but also acts as a temporal regulator during RM mobility. Specifically, C protein delays REase expression so the MTase has time to completely modify the genome, to prevent cell death due to REase cutting (17,21,22).

**Figure 1.**
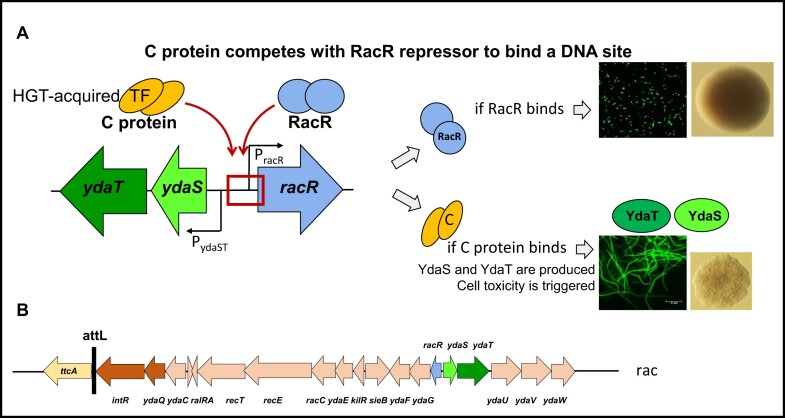
Adventitious transcriptional cross-talk occurs between two transcription factors: C protein regulating the Csp231I restriction-modification system that might be acquired by horizontal gene transfer, and RacR repressor controlling Rac prophage genes. Under physiological conditions, the RacR repressor binds within the Rac intergenic region, blocking the possible common promoter/operator region for *ydaS* and *ydaT*. As a result, *ydaS* and *ydaT* are completely silenced. When a DNA fragment carrying the C protein gene enters the cell, both transcription factors compete for binding to the same region. C protein interferes with RacR repressor binding, so *ydaST* gene expression is not sufficiently blocked. When YdaS and YdaT are produced, the likely toxicity of YdaT triggers the cell division defect (or others) and cell filamentation occurs, along with an aberrant colony morphology (**A**). Part of genetic map of *rac* locus is presented with attachment site (attL) within genomic *ttcA* gene (**B**).

During our studies, we noticed that this R-M system's C protein (C.Csp231I) also has some unpredicted side-effects, which initially were observed as inducing a toxic effect reflected in a cell morphology phenotype. We studied this phenomenon in detail using a combination of genetics, biochemistry and transcriptomics, and found that the C regulator C.Csp231I changed global *E. coli* K-12 genetic networks. In particular, we established a link between C protein binding within the Rac prophage, and cell toxicity manifested by profound filamentation (Figure 1A, B) (23,24). Our results indicated that the C protein was engaged in transcriptional cross-talk, *via* off-target DNA binding, rather than by action at its native site (target C-box) within its own promoter. By cross-talk, we mean here the adventitious binding of a TF with an unrelated (off-target, non-cognate) DNA site, sometimes called TF promiscuity (25).

This observation brought our attention to the *racR-ydaS-ydaT* locus, coding for three TFs, and its intergenic region, where the RacR master repressor competes with YdaS to bind their operators – if RacR wins this competition *ydaS* and *ydaT* expression is silenced and lysogeny is maintained (24,26,27). However, when C protein appears, RacR gene expression is derepressed, YdaS and YdaT are produced, and cell filamentation is triggered leading to cell death if these conditions are prolonged (Figure 1A) (23,24). Still, the basis for this unleashed toxicity was not known, so we designed experimental approaches to find the *E. coli* genomic target site(s) for YdaS and YdaT. As we reported before, no toxicity is detectable in an *E. coli* Δ*rac* variant (23). We hypothesized that the coexistence of C protein gene and Rac locus with RacR repressor gene creates a genetic conflict. Hence, selective pressure may lead to emergence of inactivating mutations in one component out of these two (23), which we confirm in this study.

Our main objective here was to generate *E. coli* mutant libraries, in the presence and absence of C.Csp231I-RacR conflict, to find genes involved in the toxic pathway initiated by C protein. Our results support an analogy of RacR repressor to λCI, where both proteins act as lysogeny maintenance sentinels. We also found that reduction of RacR expression is critical for Rac excision from genome and eventual loss of the prophage DNA. Rac induction appears to counteract the lethal toxicity of YdaT. Cells can apparently survive the relatively short ‘toxic’ period, until they are cured of Rac prophage. Overall, our studies on Rac prophage biology show an interesting example of a flexible strategy, in which bacteria use potent TFs to maintain beneficial prophage genes, while silencing those likely to be deleterious.

## Materials and methods

### Bacterial strains, plasmids, bacteriophages and oligonucleotides

The bacterial strains, phages and plasmids used in this study are listed in Supplementary Table S1; the oligonucleotides are in Supplementary Table S2 of the Supplementary Materials.

### Generation of Tn*5* mutant libraries

The plasmid pRL27 (28), which carries the transposon Tn*5* with a kanamycin (Kan) resistance gene, was used to transfer the transposon into MG1655*rac^+^* cells carrying the plasmid pBAD-CWT, with the C.Csp231I regulatory protein expressed under arabinose induction. A second library was prepared in MG1655Δ*rac*, with pBAD-ydaT expressing the *ydaT* gene under arabinose induction. Creation of the mutant libraries was done by conjugating the strains BW20767 and MG1655 which served as a donor and a recipient of plasmid pRL27, respectively. Briefly, 50 μl of log phase cell samples were washed away from antibiotics and inoculated simultaneously in one spot on an LB agar plate supplemented with 0.1% arabinose. After 16 h of incubation at 37°C, the whole spot was transferred to 500 μl LB, serially diluted and spread on LB agar plates supplemented with Kan, chloramphenicol (Cam) and 0.1% arabinose. Following overnight incubation formed colonies were visually inspected for the desired phenotype with a stereo microscope (Olympus).

### Transposon insertion localization

The selected Tn*5* integrants were grown in LB medium supplemented with Kan, Cam and 0.1% arabinose. Chromosomal DNA was extracted according to the manufacturer's recommendations (Roche) and digested with a restriction enzyme which does not cut within the transposon sequence of pRL27 (Supplementary Table S1). In about a quarter to one-third of the cases digestion with one enzyme (EcoRV) was sufficient to reveal products in subsequent PCR reactions, while in others a combination of two enzymes out of EcoRV, BamHI, NdeI, NheI, NcoI, ScaI was required. The resulting fragments were cleaned (A&A Biotechnology, Poland), and self-ligated using the T4 ligase (Eurx, Gdansk, Poland) at 16°C overnight. The obtained DNA fragments were again cleaned and inverse-PCR amplified by using tpnRL17-1 and tpnRL13-2 (Supplementary Table S2), which anneal to positions within the transposon sequence in pRL27 and read outwards into flanking DNA regions. To avoid amplification of non-specific fragments initial annealing temperatures were set to 74°C and were gradually reduced as specified in Supplementary Table S3 and Supplementary Table S4. Obtained DNA fragments were analyzed on 1% agarose gels to confirm random insertion. The bands that differed from the rest were cut from the gel and cleaned up (bands appearing at the same height in every path were omitted). The site of insertion of the transposon was determined by sequencing (Genomed, Poland) with the tpnRL17-1 primer. The BLAST (NCBI) and EcoCyc was used for genes identification (29). All library integrants are presented in Table 1, and Supplementary Table S5 of Supplementary Materials. Strains, for which the above-described procedure was not sufficient to identify transposon insertion site, were whole genome sequenced (DNA Sequencing and Oligonucleotide Synthesis Laboratory IBB PAS, Warsaw, Poland).

**Table 1.** Integration sites for Tn*5* mini-transposon insertions

Tn*5* mutant	Interrupted gene by Tn*5* insertion	Gene product (based on EcoCyC or Blast)	Tn*5* position (*)	Rac presence (*racR* PCR product) #
35	*kefC*	glutathione-regulated potassium-efflux system protein KefC	47769	–
93	*thiP*	fused thiamin transporter subunits of ABC superfamily: membrane components	72911	–
37	*yadN*	putative fimbrial-like adhesin protein YadN	156299	–
27C / 27B	*yaeF*	peptidase C92 family protein YaeF	216233	+
20	*fadE*	acyl-CoA dehydrogenase	242765	+
31	*phoE*	outer membrane porin PhoE	259990	+++
77	*yagE*	CP4-6 prophage; putative 2-dehydro-3-deoxygluconate aldolase	282300	–
54	operon *yagK*	operon *yagK*, CP4-6 prophage	293075	–
105	*rclB*	DUF1471 domain-containing protein RclB	318331	–
49	*yahN*	amino acid exporter for proline, lysine, glutamate, homoserine	345666	–
80	*ybaK*	Cys-tRNAPro and Cys-tRNACys deacylase	506881	+++
34	*ybdL*	methionine transaminase	633678	–
104	*ycgL*	PF05166 family peptide YcgL	1227801	–
57	*dgcJ*	putative diguanylate cyclase	1872041	–
8	*rcsA*	DNA-binding transcriptional activator, co-regulator with RcsB	2023968	+
48 / 108	*wcaD*	colanic acid synthesis	2130451	–
36	*arnB*	UDP-4-amino-4-deoxy-L-arabinose aminotransferase	2366760	+
40	*pdeA*	putative c-di-GMP phosphodiesterase PdeA	2516100	+++
55	*csdE*	sulfur acceptor protein CsdE	2944849	–
68	*yqeG*	putative transporter YqeG	2986980	–
97	*yqeL*	uncharacterized protein YqeL	2989819	–
42	*aaeR*	LysR-type transcriptional regulator AaeR	3389668	–
103	*yiaL*	DUF386 domain-containing protein YiaL	3743743	–
46	*waaH*	UDP-glucuronate:LPS(HepIII) glycosyltransferase	3789047	–
29	*waaZ*	lipopolysaccharide core biosynthesis protein WaaZ	3800147	+++
61	*waaG*	lipopolysaccharide glucosyltransferase	3806442	–
67	*waaQ*	lipopolysaccharide core heptosyltransferase 3	3807472	–
63	*waaQ*	lipopolysaccharide core heptosyltransferase 4	3807482	+
47	*rirA*	small regulatory RNA RirA (RfaH interacting RNA)	3808201	+
81	*nlpA*	lipoprotein-28	3839916	–
4	*rbsK*	ribokinase	3937294	+++
83 / 94	*cyaA*	adenylate cyclase	3991182	+++
78 / 17	*arpA*	regulator of acetyl CoA synthetase	3937294	–
18	*phnC*	phosphonate/phosphate ABC transporter ATP binding subunit	4324426	+
53	*dnD*	L-idonate 5-dehydrogenase, NAD-binding	4493375	–
91	*yjhI*	KpLE2 phage-like element; putative DNA-binding transcriptional regulator YjhI	4525231	+
100	*yjhZ*	putative acetyltransferase YjhZ, KpLE2 phage-like element	4534545	–
84	*idlP*	iraD leader peptide	4556938	+++

(*) indicates position relative to the *Escherichia coli* K-12 substr. MG1655, version 26.5 on the website www.ecocyc.org convergent with U00096.3.

(#) indicates intensity of PCR product: weak (+), strong (+++), or no product (–).

### Complementation of the Tn*5* mutation

To confirm that the obtained defect in cell morphology is due to C protein/YdaT expression and it is blocked by inactivation of certain genes selected from Tn*5* library, these selected genes were cloned as the intact genes under an inducible promoter. Cloning vector pHM1786 contains the lacI^q^ repressor gene, and the IPTG (isopropyl β-D-1-thiogalactopyranoside) inducible P_tac_ promoter (Supplementary Table S1). Both the vector and the PCR-amplified insert were digested with HindIII and EcoRI, ligated and transferred to the cloning strain. Positively verified recombinant plasmids were used in the complementation assay. The assay included two compatible plasmids: one with the intact selected gene (pHM1786 derivative), and the second with an inducible form of the gene for either C.Csp231I (pBAD-CWT) or YdaT (pBAD-ydaT). These two plasmid pairs (and their empty vectors as control) were introduced into the Tn*5* insertional mutant strains under antibiotic selection and gene induction (IPTG, arabinose). The fresh transformant cells were examined under a microscope for the filamentous phenotype.

### Chromosomal gene knock-outs

The knockout strains were constructed using the lambda-red recombination method with a pSIM5 plasmid carrying the recombineering proteins, Gam, Exo and Beta (30,31), and using pKD46 as a template plasmid for ampicillin resistance cassette amplification. The constructed strains and primers used are listed in Supplementary Table S1 and Supplementary Table S2 (Supplementary Materials).

### Assay for excision of the Rac prophage

Three types of MG1655*rac*^+^ strain were tested with *bla* cassette (ampicillin (Amp) resistance gene) inserted into the *rac* locus: within *ydaS*, *ydaT* or *ralRA*. Each strain was transformed with pBAD-CWT or pBAD-Cmut (non-DNA-binding C protein variant, described in (18)). The *ydaT* expression (pBAD-ydaT plasmid) or *ydaS* expression (pBAD-ydaS) was also tested for effects on Rac excision. Transformants were subcultured every 10 generations in the presence of 0.1% arabinose over a period of five consecutive days, and samples were spread onto Cam LB-agar plates (without Amp or arabinose). A hundred colonies per strain were streaked in parallel on both LB-agar and LB-agar Amp plates. After overnight incubation colonies were inspected for the selective lack of growth in the presence of Amp. Colonies were counted in duplicate and data presented in percentage of total colonies tested. In addition, colonies were also examined for the filamentous phenotype.

### Quantification of prophage excision

*E. coli* MG1655 *rac*^+^ cells, carrying the plasmid with C protein WT gene (or Cmut, unable to bind DNA (18)) under control of the inducible P_BAD_ promoter, were grown overnight in LB medium with glucose and appropriate antibiotics. After dilution, the two cultures were grown in LB-glucose to an OD_600nm_ of 0.4, then cells were gently pelleted, washed, and split into three replicate cultures. Pre-induction samples were taken (generation 1), then the LB was supplemented with 0.1% arabinose and cultures were left for continuous sub-culturing under the induced conditions (samples were taken up to generation 50).

The prophage excision triggered by C protein expression was quantified by PCR (qPCR). Two sets of primers were selected. One pair (ttcAqPCR, intRqPCR; Supplementary Table S2) flanked the region between the Rac left attachment site (*ttcA* gene in chromosome) and the first gene within the Rac prophage (*intR*) (amplicon 216 bp) (schematic location of primers; Supplementary Figure S1). The second set of primers amplified part of the *ydaS* and *ydaT* genes (primers ydaSpLEX3Bfor, ydaTpLEX3Brev, 183 bp amplicon). For normalization, two alternative housekeeping genes were used: *idnT* (gluconate transporter; idnTfor, idnTrev, 200 bp amplicon; Supplementary Table S2), and *zntB* (Zn^2+^/H^+^ symporter, 2865 bp from the *ttcA* start; primers zntBqPCRfor, zntBqPCRrev, 129 bp amplicon). Total DNA was isolated using a High Pure DNA isolation kit (Roche) and was used as the template for the qPCR reaction using the SG qPCR Master Mix (Eurx, Poland). The qPCR conditions were as follows: pre-denaturation step 95°C, 5 min and 30 cycles of 95°C for 10 s, annealing 56°C for 10 s and elongation at 72°C for 10 s. The reaction and analysis was performed using the Roche Light Cycler. Each reaction was performed in biological triplicates and repeated at least twice independently. Data were averaged and standard deviation was calculated. Melting curve analysis was used to confirm the formation of the specific products. The calculations also included the PCR efficiences and its C_t_ was plotted against DNA input to calculate the slope corresponding to PCR efficiency obtained with high linearity (*R*^2^> 0.97) (32–34).

### Determination of transcript levels by q-RT-PCR

One ml of exponentially grown *E. coli* MG1655*rac*^+^ cells on LB-arabinose medium, harboring plasmids carrying: the inducible C gene (pBAD-CWT), the inducible RacR gene (pBAD-RacR) or empty vector (pBAD33), were harvested. The bacterial pellet was resuspended in 1 ml of StayRNA reagent to prevent RNA degradation (A&A Biotechnology). The total cellular RNA was then extracted using the Total RNA Mini Plus kit (A&A Biotechnology) according to the manufacturer's instructions. After elution, the RNA was treated with DNaseI (A&A Biotechnology) for 60 min at 37°C following enzyme inactivation for 15 min at 65°C. The RNA was then used to synthesize first-strand cDNA using RevertAid First Strand cDNA Synthesis Kit (Thermo Scientific). Several sets of primers (Supplementary Table S2) were used to estimate transcript levels in qPCR reactions (Roche Light Cycler 480), specific to Rac genes (*intR*, *racR*, *ydaQ*) with *idnT* being used as the reference gene. Each qPCR reaction (25 μl) contained 12.5μl SG qPCR Master Mix (2×) with SYBR Green I fluorescent dye, Perpetual Taq DNA polymerase and dNTPs (Eurx Poland), 7.75 μl H_2_O, 0.25 μl of UNG (Uracil-N-glycosylase), 1 μl of 10μM forward and reverse primers mix and 2.5 μl of diluted cDNA as a template. The qPCR conditions were as follows: UNG pre-treatment 50°C for 2 min, pre-denaturation step in 95°C for 10 min and 35 cycles of 94°C for 15 s, annealing 55°C for 30 s and polymerization at 72°C for 30 s and final acquisition 80°C for 15 s. The expected sizes of products were between 171 and 200 bp. Each reaction was performed in biological triplicates and repeated at least twice independently. Data were averaged (±SD) and Student's *t*-test was calculated. Melting curve analysis was used to confirm the formation of the specific products. The calculations also included the PCR efficiencies, where each cDNA was serially diluted and its Ct was plotted against cDNA input to calculate the slope corresponding to PCR efficiency obtained with high linearity (*R*^2^> 0.98) (32–34).

### Sequencing and genome analysis

Genomic DNA was extracted from cells using Genomic Midi AX (A&A Biotechnology) according to the manufacturer's instructions. DNA was ethanol-precipitated and resuspended in TE buffer. DNA was separated in agarose gel to assess its high quality (no observed degradation). Samples were diluted to achieve *A*_260_/*A*_280_ ratio in the range 1.8–2.0 with concentrations above 10 ng/μl.

For whole-genome sequencing, paired-end whole-genome next-generation sequencing (NGS) was performed on Illumina Sequencing PE150 (Novagene, UK), with read lengths of 150 bp. The raw reads were subjected to quality control with FastQC and due to overall good quality were not processed further. Next, reads were aligned to the reference genome *E. coli* K-12 MG1655 (GenBank assembly accession GCA_000005845.2) using BWA alignment software, version 0.7.17-r1188 (35). Mapping quality was assessed using QualiMap version 2.2.1 (36). The latter allowed identification of regions with larger genomic interruptions, that were further verified manually and visualized using JBrowse2 (37). Data were deposited in the NCBI (bioproject accession number PRJNA993445).

An RNA-seq library was prepared according to the TruSeq RNA Sample Preparation, version 2 Guide (Illumina, San Diego, CA, USA) and sequencing of the libraries was performed using an Illumina HiSeq2500 platform at Macrogen. The results have been published (23) and raw data deposited in the NCBI GEO (accession number GSE126248).

### The *ydaT and ydaS* toxicity assay

An overnight culture of *E. coli* BL21(DE3) carrying two compatible plasmids, p51ydaT (IPTG-inducible *ydaT* expression) and pBAD-ydaS (arabinose-inducible *ydaS* expression), was inoculated into LB containing 100 μg/ml Amp and 34 μg/ml Cam, grown to early log phase (OD_600nm_ ∼0.2), followed by induction of gene expression with either 1 mM IPTG, 0.1% l-arabinose, or both 1 mM IPTG and 0.1% L-arabinose. Control cultures remained uninduced, treated with glucose. Bacteria were further grown with shaking for additional 3 h, and the samples were taken at indicated time points and after serial dilution spotted on LA containing antibiotics and appropriate inducer. Other *E. coli* strains were tested at identical way.

### Overproduction, purification and size-exclusion chromatography of the RacR repressor

For the C-terminally-6His-tagged RacR overproduction and purification were performed as reported in Supplementary Materials in (24). Oligomerization status was tested by size exclusion chromatography on a Superdex 75 10/300 GL column using the ÄKTA Pure 25 system (GE Healthcare). The predicted size for monomer of 6His-tagged RacR repressor is 18.5 kDa. The 0.5 mg protein sample was loaded onto the column equilibrated with 50 mM potassium phosphate buffer (pH 7.0), 150 mM NaCl, and eluted at a flow rate of 0.5 ml/min in the same buffer. The column was calibrated with proteins of known molecular masses: alcohol dehydrogenase (tetramer), 146.8 kDa; bovine serum albumin, 66 kDa; ovalbumin, 43 kDa; trypsin inhibitor, 22 kDa; and cytochrome C, 12.4 kDa (Sigma-Aldrich, St. Louis, MO, USA).

### Bacteriophage spot assay

To determine the efficiency of phage lytic productivity, virulent bacteriophage samples (P1_vir_ and λ_vir_) were used. Log phase BW25113 *rac*^+^ bacteria cells carrying the pBAD-YdaT or pBAD33 were 0.1% arabinose induced for 2 h, and 30 μl samples were mixed with 10 μl phage samples, serially diluted in TM+ buffer (10 mM MgSO_4_, 10 mM TRIS and 0.01 mM CaCl_2_). After 10 min incubation time at 37°C, successive dilutions were spot plated (5 μl) using LB-agar with Cam and incubated overnight at 37°C. Bacterial colony forming units (CFU) without phage addition were checked as control.

### Bioinformatics analysis and molecular modelling

The 3D structures of RacR was predicted utilizing a range of tools: AlphaFold2 (38), ESM (39), OmegaFold (40), RGN2 (41), Phyre2 (42), HHsearch (43) and Modeller (44). Comparative analysis, structural alignment, and modeling part were conducted using UCSF Chimera (45). Putative oligomers were identified through AlphaFold-Multimer and GalaxyHomomer (46). DNA-protein interaction was predicted using HDOCK (47) and *via* comparison with DNA-containing templates from homologous structures (e.g. λCI repressor) in the PDB, identified by FoldSeek (48).

The files related to the bioinformatics protein modelling are accessible at RePOD repository: https://doi.org/10.18150/FV9R0P

### Statistical analysis

Unless otherwise indicated, we performed statistical analysis and presented plot data as the averages and standard deviations. For column plots, data were analyzed using GraphPad Prism 8. Student's *t* test was performed to calculate a p value for the difference between related pairings (***P < 0.001; *P < 0.05 and ns: *P* > 0.05).

## Results

### Screening and analysis of the Tn*5* insertion mutant library

In order to identify the *E. coli* genetic loci participating in the C.Csp231I protein-dependent cell filamentous phenotype (presumably mediated by the YdaS and YdaT gene products) (Figure 1), we used an unbiased, functional approach. We generated a Tn*5* transposon random insertion library of *E. coli* MG1655 cells, employing a vector carrying a mini-Tn*5* transposon (Kan^R^) and a modified hyperactive version of the gene coding for the Tn*5* transposase (pRL27) (28). In this library, after induction of C protein expression all colonies display a distinct flat, abnormal curly morphology (Figure 1). In theory, if a gene responsible for the C-dependent phenotype was interrupted by Tn*5* – leading to loss of the filamentation then supplying a WT copy of that gene should restore the filamentous phenotype. This morphological difference enables us to easily distinguish standard colonies among the majority of morphologically-abnormal colonies under a magnifying glass, or even by eye alone.

**Figure 2.**
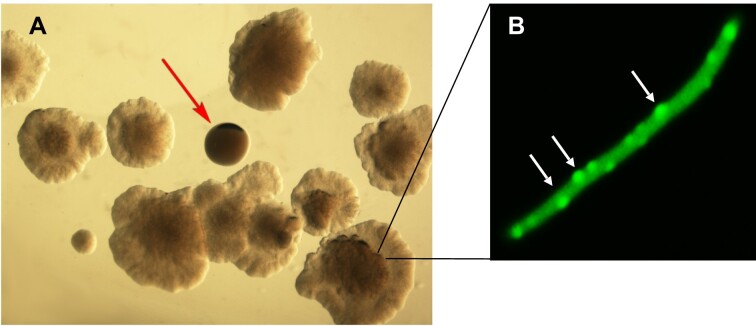
Screening the Tn*5* insertional mutant library. The vast majority of colonies showed an abnormal morphology, with a flat center and irregular, swarming-like boundary, once C protein expression was induced. The objective was to find normal colonies (shown by red arrow), where the ‘sick’ colony phenotype was suppressed. (**A**) A representative image is shown, acquired by Olympus stereo microscope. (**B**) To visualize the Z-ring prior cell division from ‘sick’ colony, cells with two plasmids carrying inducible C protein and fusion protein FtsW::GFP were grown under arabinose and IPTG induction. A single filamentous cell containing several randomly distributed spots of FtsW::GFP (white arrows).

The representative *E. coli* MG1655 Tn*5* insertional mutant library was created on agar plates with Kan (selecting for Tn*5*), Cam (selecting for the *csp231IC* plasmid), and arabinose (to induce C protein production). Inspection of the plated library revealed the expected defective colony morphology in the great majority of colonies (Figure 2A). The prepared library, as two technical replicates, each consisted of about 8 × 10^5^ integrants, which were screened thoroughly to find ‘regular’, opaque colonies, as shown by red arrow in Figure 2A. Such Tn*5* mutants were isolated, restreaked in sectors, and examined under a microscope to ensure that they were filament-free. Forty-one mutants were isolated and frozen for further studies. In total, we found 108 colonies with normal morphology, among 1.6 × 10^6^ colonies screened, for a hit rate of 6.75 × 10^−5^ (the total ratio of the number of normal to filamentous colonies). Filamentous cells were on average between 15 to 25μm in length and consisted of several segments with visible DNA inside (23). Such cells formed the majority of defective colonies, which seemed to have flawed septum formation during cell division, as indicated by scattered distribution of FtsW protein, which assists in Z-ring assembly (49,50) (white arrows on Figure 2B using FtsW::GFP, pDSW360).

**Figure 3.**
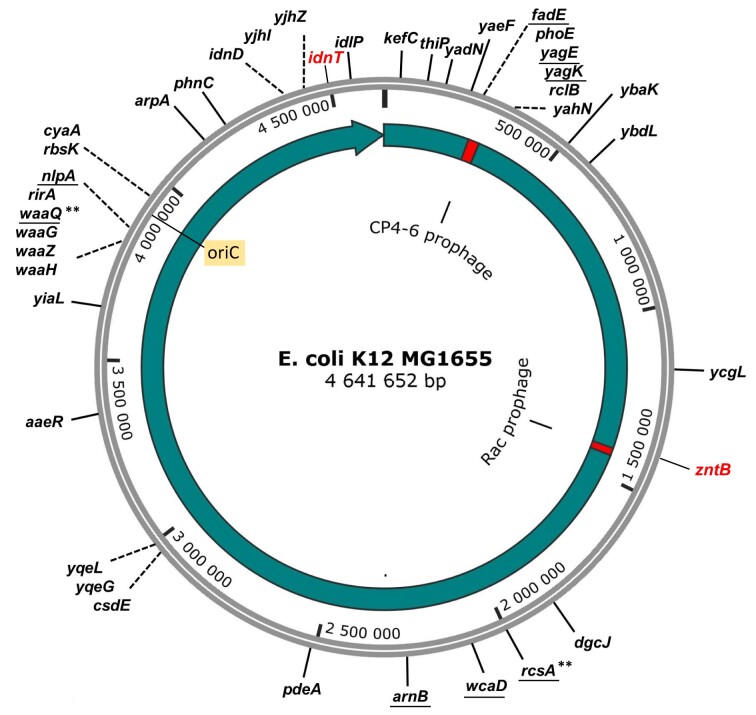
Genomic map of *E. coli* MG1655 showing identified genes interrupted with Tn*5* transposon insertions. Rac prophage and CP4-6 prophage regions indicated by red sectors. The positions are taken from the reference genome *E. coli* MG1655 (GenBank U00096), and are also shown in Table 1. Underlined are genes used in the complementation assay. For two genes: *waaQ* and *rcsA*, a directed knock-out was made (indicated by **). Genes used for normalization of DNA level: *zntB* and *idnT* are marked in red. Origin of replication (oriC) is also indicated in box.

Next, the locations of Tn*5* insertions within the selected group of mutants was determined by Tn*5* subcloning and inverse-PCR (28), as described in Methods, Supplementary Figure S2, and Supplementary Tables S3, S4. This approach worked well for most mutants, however for a few of them we performed whole genome sequencing using *E. coli* MG1655 as the reference strain control. The site of Tn*5* integration was unique for each mutant isolated (Table 1, Supplementary Table S5 and Figure 3). DNA sequence analysis showed that Tn insertion had no particular hot-spots. Nevertheless, some genes were disrupted in several distinct positions (Figure 3), such as *wcaD* (colonic acid polymerase), *cyaA* (adenylate cyclase) and *arpA* (a regulator of acetyl CoA synthetase). Five insertions were within the *waa* operon, involved in assembly of the core region of the lipopolysaccharide, which stabilize the cell's outer membrane (Table 1, Figure 3). There were also some insertions within TF genes, such as *rcsA*, which controls a phosphorelay system; *aaeR*, which is a LysR-type regulator; and *yjhL*, which regulates phage-like genes. A few genes within the cryptic CP4-5 prophage region were also disrupted: *fadE*, *phoE*, *yagE*, *yagK*, *rclB* and *yahN* (Figure 3). Many identified interrupted genes are involved in sugar metabolism or transport. For some strains with interrupted genes such as *waaG*, *waaQ* and *rirA*, the cells revealed a mucoid, shiny colony phenotype.

To confirm that the filamentous and colony phenotypes of cells depends on C protein expression in our system, and to rule out polar effects, we performed complementation assays. Introducing the respective intact gene *in trans* on a plasmid should revert the phenotype from normal to ‘sick’ colony morphology, in the strain where the tested gene is interrupted by Tn*5*. Thus, we cloned selected genes (from Table 1) under an inducible promoter. We used the following genes: *arnB, fadE, nlpA, rcsA, waaQ, wcaD, yagE* and *yagK*. For all of them, co-expression with C protein gene in the genetic context of Tn*5*-inactivated genes did not change the cell morphology significantly. So, the complementation assay was not successful under our conditions. The reasons for this are described in the remainder of this section and the following one.

We next tested whether the revealed mutants are C protein responsive. Hence, we made a directed gene knock-out for two genes: *waaQ* (involved in lipopolysaccharide synthesis (51,52), which appeared twice in the library) and *rcsA* (a multifunctional transcriptional activator involved in biofilm production, cell division, motility, swarming and other functions (53,54)). Next, we introduced the plasmid specifying C.Csp231I and found out that these cells remained normal and failed to filament despite C protein overexpression (Supplementary Figures S3, S4). This result indicated that the library had indeed yielded mutants (at least *waaQ* and *rcsA*) associated with resistance to C protein overexpression, even though the complementation results are unclear.

In addition, we knew that induction of C protein directly affects the Rac genes based on our transcriptomic data analyzed previously (23,24). Accordingly, we expected some Tn*5* insertions in the Rac region itself, but surprisingly there were none among the library mutants we characterized (Figures 1B and 3, Table 1). This fact made us address the issue of Rac presence in the library genomes. Thus, we first decided to test some Tn*5* mutants for the presence of the Rac *racR-ydaS-ydaT* region, using PCR amplification with lysed frozen cells samples as a reaction template. In most cases, we obtained very weak or undetectable amplification products (Table 1 and Supplementary Figure S5). We concluded that deletion within the Rac region might have occurred, or possibly prophage excision. In addition, we analyzed the whole-genome sequence of selected mutant DNAs, and observed the larger deletion within Rac locus.

### Insufficient RacR repressor level is critical for rac excision

**Figure 4.**
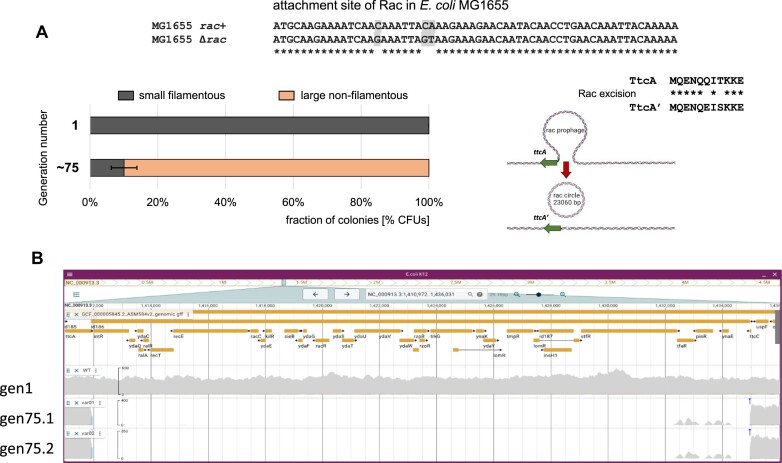
Rac prophage excision from the *E. coli* K-12 MG1655 genome is triggered by C protein expression. (**A**) The nucleotide sequence of the left and right attachment sites of Rac within the host *ttcA* gene before and after Rac induction. Excision of Rac into a DNA circle also results in changes of amino-acid residues in the TtcA protein (Q6E, T8S), as shown. Prolonged *rac*^+^ cells exposure for C protein expression induced a selective pressure for *rac*^+^ cells to lose the filamentation phenotype. Initially all cells were filamentous and formed uniformly small colonies, but after passaging for ∼75 generations only ∼10% of cells had this phenotype. The remaining 90% of cells spontaneously changed to producing large colonies containing normal, rod-shaped cells. (**B**) Nine such colonies were grown, their chromosomal DNA was isolated, sequenced, and compared to the genome sequence from generation 1. All nine genomic sequences under comparison revealed identical deletion of the Rac prophage from the attachment site, as presented in the top sequence for Δ*rac*. Control genomes contained the entire WT *rac* locus.

To further verify the role of Rac prophage genes, and especially of the *racR-ydaS-ydaT* operon (Figure 1B), in the C-protein-responsive phenotype, we tested whether cells lose the filamentous phenotype spontaneously after long exposure to C protein expression. We subcultured MG1655 *rac*^+^ cells carrying the p24 plasmid (with *csp231IC* under its natural promoter) for many generations, by daily passaging, as we had done earlier (23). About 75 cell generations produced almost 90% colonies with round and regular shapes, losing the initial phenotype (Figures 1, 2B and 4A). We took nine such colonies and one control colony (prior to p24 plasmid introduction), isolated their genomes and subjected all 10 to whole genome sequencing. We expected to find some suppressing mutations, which neutralized the YdaS/YdaT toxicity. Indeed, we found one common mutation among all nine tested genomes, that was not present in the control genome (Figure 4B). We detected three base changes within the 5′ end of *ttcA* (involved in post-transcriptional tRNA thiolation). Significantly, this locus is the specific integration/excision site for the Rac prophage (55). Close inspection of this region revealed identical 23060 bp deletions of the Rac prophage in all nine tested genomes (Figure 4B). The analysis revealed the same specific point mutations as had been reported previously, during studies on Rac excision in *E. coli* K-12 BW25113 cells after the overproduction of the Rac excisionase (*ydaQ*= *xisR*) and integrase (*intR*) proteins (55). Thus, both assays, utilizing our Tn*5* library, as well as the spontaneous mutants, suggest a common selective pressure, favoring Rac prophage excision to block prophage gene expression, apparently including the *ydaS/ydaT* genes. This finding explains why our complementation assays did not succeed – after prolonged cell growth with C overexpression, Rac prophage undergoes induction, and is subsequently lost, along with expression of the potentially toxic genes. This cell propagation might have happened as recently as during preparation of competent cells from Tn*5* mutants for the complementation assays. Our observation would also explain the accumulation of morphologically normal colonies, as these strains had lost the Rac prophage (including *ydaS/ydaT*). In addition, it is possible that some of the Tn*5* integrants had lost Rac before Tn*5* integrated, and were actually false mutants, that played no role in the filament forming phenotype, though this is clearly not the case for *waaQ* or *rcsA* (see above, and Supplementary Figures S3 and S4).

### Kinetics of rac excision in the presence of C protein over-expression

Our previous whole-transcriptome study (23) showed that several genes in the Rac prophage are significantly activated in C protein-expressing cells, as compared to C-absent cells. The Rac genes showing increased expression included: *ydaS*, *ydaC*, *kilR, ralA, ydaF, ydaG, ydaE* and *racC* (ranging from 40- to 70-fold change) (Figure 1B). Hence we did not expect to see Rac prophage excision and circularization.

**Figure 5.**
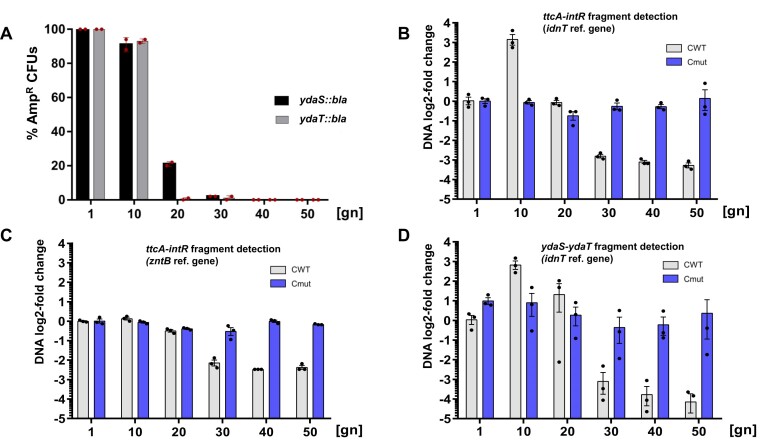
Kinetics of Rac prophage excision from *E. coli* K-12 genome. (**A**) Phage excision was measured as cell growth on Amp-supplemented LB-agar plates. Two derivatives of *E. coli* MG1655*rac*^+^ were tested, where an Amp-resistance cassette (*bla*) was inserted into one of two Rac genes, yielding *ydaS::bla* or *ydaT::bla*. These cells, carrying a plasmid with the C protein gene *csp231IC* controlled by the inducible promoter P_BAD_, were incubated in glucose-LB (generation 1). A small fraction was gently pelleted, washed and supplemented with 0.1% arabinose-LB for continuous sub-culturing (up to generation 50). At each indicated time, isolated colonies were picked and streaked onto LB-agar plates with and without Amp in duplicate, and the % of resistant colonies was determined. (B–D) Relative levels of Rac DNA after induction of C protein were also determined using qPCR (see Methods) on isolated total DNA from the same assay described in (A). However, *E. coli* MG1655*rac*^+^ cells were used with induced C protein WT or its non-DNA binding mutant, Cmut, as a negative control. (**B**) First we used the DNA segment for amplification that spans the Rac attachment site, using primers matching genes from both sides: *ttcA* on genomic side and *intR* on the Rac side. For normalization of DNA amounts, the housekeeping *idnT* gene was used. (**C**) The same samples were analyzed using different normalization primers, within the *zntB* gene located much closer to the Rac attachment site than *idnT*. (**D**) Additional measurements were also taken for a DNA segment amplified from the middle of Rac, within *ydaS/ydaT*, again using *idnT* for normalization. Statistical analysis were performed as indicated in Methods, each reaction was performed in biological triplicates. Averages and standard deviations are shown. Comparisons between column bars for CWT for 1st versus 50th generation number indicate significant difference, with a *P* value = 0.0025 (panel B); *P*< 0.0001 (panel C); *P* = 0.0042 (panel D).

In light of our studies here with the Tn*5* libraries, it is significant that certain stresses result in Rac DNA excision (55). We sought to find out how quickly this process occurs. To monitor the Rac excision over time, we first used a PCR approach, growing cells with the plasmid carrying the C protein gene under the inducible P_BAD_ promoter (pBAD-CWT). We observed that Rac induction did not take place within 15 h after adding arabinose. Thus we decided to monitor this process over many cell generations. In the first approach, we used the two genomic MG1655 mutants: *ydaS::bla* and *ydaT::bla*, where the respective Rac genes *ydaS* and *ydaT* were interrupted with a *bla* cassette (Amp^R^). Specifically, these strains grow on Amp plates only when Rac is stably maintained in the genome, while Rac excision would eventually lead to loss of resistance, as the cells would not express Rac genes, including the *bla* cassette. Next, we introduced plasmid pBAD-CWT with the C protein gene. The resulting transformants were induced with arabinose for continuous C protein expression, and sub-cultured every ∼10 generations for up to 5 days in liquid culture. Single colonies were isolated and screened on Amp plates. Overall, the results showed that phage excision is not a rapid process, as during the first 10 generations the number of Amp-resistant colonies hardly changed (Figure 5A). However, after that a sharp decline in number of such cells is observed, reaching nearly no Amp-resistant colonies at generation 30. These results are consistent with earlier data showing lower viability of cells with C protein expressed only in *rac*^+^ strain, but not in Δ*rac* (23). In addition, this observation explains also why our whole-transcriptome analysis have shown Rac genes’ expression (23), as cell samples for RNA-seq were taken from fresh transformants, within the generations, where Rac excision was not detected.

The implied excision and loss of Rac DNA during C protein expression prompted us to investigate this process in a more sensitive manner, using qPCR analysis. We used cells with C protein (CWT), or as a negative control its mutated inactive variant (Cmut = A33G; R34E; Q37A, which is unable to bind its specific DNA; pBADCmut (18)) (Figure 5B–D). We employed two pairs of primers: (i) flanking the Rac attachment site, within genomic *ttcA* and Rac *intR* genes (Figure 5B, C, Supplementary Figure S1); and (ii) in the center of Rac, within *ydaS* - *ydaT* (Figure 5D) using a primer pair within the distant host gene *idnT* for normalization (Figure 1B; Figure 3). Both assays revealed a similar time course for the relative Rac DNA levels, with a surprising initial increase up to 9-fold within the first 10 cell generations, which was not seen in the context of the mutated C protein (Figure 5BD). After 20 generations passed, the Rac excision was highly visible, as the DNA amplification product was weak (reduced by 8-fold). The control with Cmut remained stable throughout the entire course of the experiment. Next, we changed the normalization gene for the same DNA samples, from *idnT* to *zntB*, which is located much closer to the attachment site, though still on the genomic flank (∼2000 bp from *ttcA* start). We saw complete loss of the apparent increase of Rac copies (Figure 5C).

The initial difference of Rac copies compared to genome copies is not clear, as the attachment site and Rac DNA circle have been characterized before. Still, this observation was consistent and repeatable, using different pairs of primers within Rac. We tried also to monitor the Rac induced DNA circles by PCR, but with no success (Supplementary Figure S6). It is not known how deficient the Rac replication process is, but other lambdoid phages use rolling circle replication to produce one long concatameric molecule with many copies of the phage genome (56), and this may explain our observed peak of Rac copies (Figure 5B, D).

### RacR acts as a sentinel for Rac prophage maintenance

**Figure 6.**
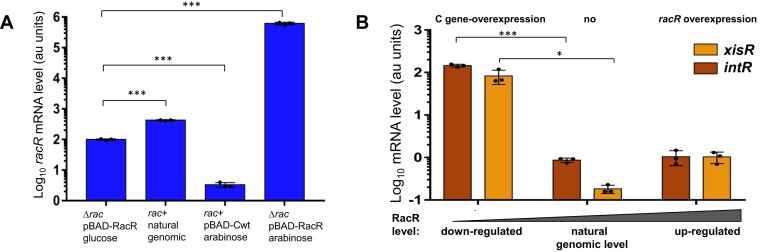
The RacR repressor depresses expression of the Rac prophage *xisR* excisionase and *intR* integrase. (**A**) Relative mRNA levels for *racR* in response to overexpression of C protein. As a control, expression of plasmid-borne *racR* (pBAD-RacR) is also shown under conditions of gene expression inhibition (glucose) or induction (arabinose). (**B**) The relative transcript levels of two genes required for Rac induction, IntR integrase and XisR excisionase. These were analyzed over a range of RacR levels. We used three conditions with different RacR expression levels: high expression (*in trans* from pBAD-racR plasmid), through medium natural genomic level (wild-type genomic *racR* and empty vector pBAD33), to low level where RacR is repressed by C protein (from pBAD-CWT). Total RNA was isolated from cells of *E. coli* MG1655*rac*^+^ carrying indicated plasmids after 2h of 0.1% arabinose induction. Statistical analysis for q-RT-PCR in panel A, B were performed as indicated in Methods, each reaction was performed in biological triplicates. Averages and standard deviations are shown. Student's t-test was performed *** *P*< 0.0001; * *P*= 0.0099.

To study further the direct effect of RacR as a master regulator of Rac induction, we analyzed the mRNA levels of two crucial proteins responsible for prophage excision: Rac excisionase (YdaQ = XisR) and integrase (IntR) (55). These genes are adjacent, and lie down next to the *rac* attachment site, but far away from RacR repressor (Figure 1B). We aimed to measure their transcript levels in response to a gradient of RacR repressor concentrations: from high expression (*in trans* from a pBAD-*racR* plasmid), through the medium natural genomic level (chromosomal Rac plus empty vector pBAD33), to the low level where RacR expression is repressed by C protein (from pBAD-CWT). Control experiments have shown that C gene overexpression inhibited genomic *racR* expression by over 100-fold, while arabinose induction of *racR* expression from a plasmid was over 1000-fold (Figure 6A). In the presence of these three RacR levels, the relative level of *xisR* and *intR* increased about 100-fold only when the RacR level was reduced relative to its natural level (Figure 6B). In contrast, the natural RacR level was sufficient to maintain optimal *xisR* and *intR* levels, and increased *racR* expression did not change their expression.

**Figure 7.**
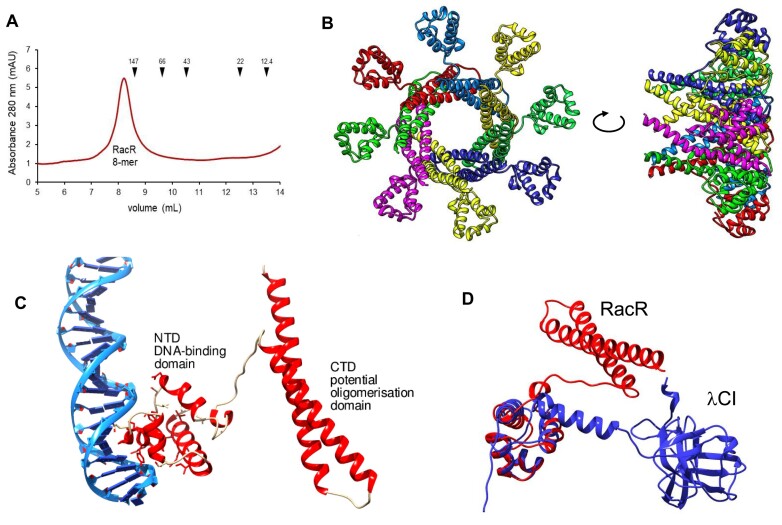
The RacR repressor exhibits two distinct domains and forms an octamer in its active state. (**A**) Size-exclusion chromatography elution profile of 6His-tagged RacR (monomer = 18.5 kDa) on a Superdex 75 10/300 GL column (GE Healthcare) supports the octameric structure. The RacR peak at 151 kDa aligns with the predicted mass of the octamer (148 kDa), indicating a close fit to the curve. Molecular mass markers are indicated by arrows. (**B**) Putative octameric structure as predicted by the HDOCK tool. (**C**) The RacR protein model, generated by the ESM program, reveals two distinct domains: the N-terminal domain, responsible for DNA binding, and the C-terminal domain, functioning as the scaffold for oligomerization. (**D**) Comparative analysis of bioinformatics models for RacR (red) and the λCI repressor (blue) reveals the high structural similarity of their N-terminal domains despite low sequence identity. Conversely, the C-terminal domains exhibit complete dissimilarity, but serve a shared purpose as the platform for oligomerization.

Previously, we hypothesized that RacR repressor may function in analogous manner as CI repressor of λ phage. We had determined RacR recognition sites within the *racR* upstream region, and found four inverted repeats as potential operators (24). Now, we tested whether purified RacR protein can form oligomers as λCI does. Using the size-exclusion chromatography approach, with co-chromatographed molecular size markers, we confirmed that RacR forms octamers, which is consistent with number of recognition sites and bioinformatics predictions (Figure 7A, B). It seems likely that each of the four inverted repeats is bound by a RacR dimer. The 3D structure prediction reveals that the RacR protein consists two distinct domains (Figure 7C and Supplementary Figure S7). The N-terminal domain (NTD) is responsible for DNA binding. Despite limited sequence identity with the CI repressor (NP_415874.1 versus P03034.2), the NTD demonstrates a similar 3D fold, composed of five helices, with the α3-helix (residues 38–47) interacting with the major groove of DNA (Figure 7C and Supplementary Figure S8). Notably, while the C-terminal domain (CTD) of RacR and the CI repressor exhibit entirely different 3D folds (Figure 7D), they serve a common purpose: facilitating oligomerization. This suggests that the RacR repressor behaves analogously to the CI repressor of the phage.

### YdaT, but not YdaS, activity reduces cell viability, and YdaT has no effect on Rac excision

**Figure 8.**
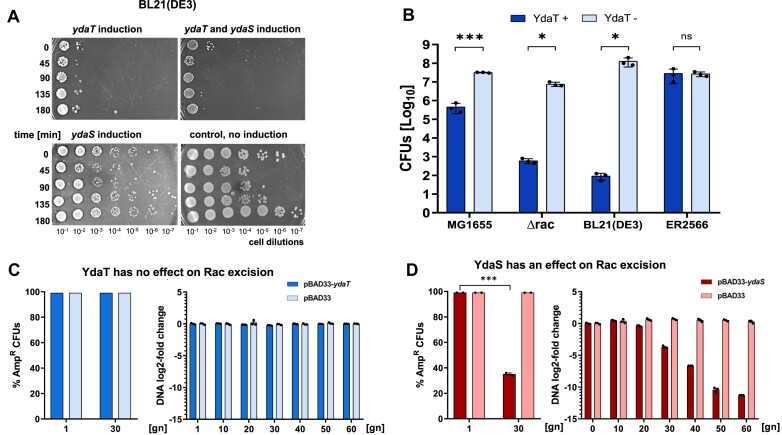
YdaT, but not YdaS exerts cell toxicity. Only YdaS affects Rac excision. (**A**) Effect of overexpressing *ydaT, ydaS* or both genes on growth of *E. coli* cells. Culture of *E. coli* BL21(DE3) carrying two compatible plasmids: p51ydaT (IPTG-inducible *ydaT* expression) and pBAD-ydaS (arabinose-inducible *ydaS* expression). These were induced with 1 mM IPTG and/or 0.1% L-arabinose, and control cultures remained uninduced, treated with glucose only. Samples were collected at indicated times after induction, and spotted onto LB-agar supplemented with Amp, Cam and the appropriate inducer. (**B**) The same approach was used to compare the growth reduction in selected *E. coli* strains. CFU’s (colony-forming units) ratio (CFU_ydaT_/CFU_vec_) of arabinose-induced cells carrying plasmid with *ydaT* gene (pBAD-ydaT) versus cells with empty vector (pBAD33) was measured in the indicated strains: *E. coli* K-12: MG1655*rac*^+^ and MG1655*Δrac*; *E. coli* B: BL21(DE3) and ER2556. Averages and standard deviations are shown. (**C**) YdaT does not affect Rac excision. YdaT effect was measured as a cell survival ratio in an experiment identical to that described in Figure 5A, where strain MG1655 *ydaT::bla* was exposed on Amp over a range of YdaT concentrations. (**D**) The same experiment was conducted for YdaS. Rac excision was measured in *E. coli* MG1655*rac*^+^ under YdaT induction, using qPCR under identical conditions, as noted in Figure 5B with housekeeping *idnT* gene as a reference gene. Statistical analysis for q-RT-PCR in panel C, D were performed as indicated in Methods, each reaction was performed in biological triplicates. Averages and standard deviations are shown. For all panels Student's *t*-test was performed with values: *** *P*<0.0001; * *P*<0.05 and ns: *P*>0.05.

The Rac excision results obtained using *E. coli* strains MG1655 *ydaS::bla* or *ydaT::bla* (Figure 5A; with inactive *ydaS* or *ydaT* genes respectively) made us re-consider the *ydaST* effects on cell viability. We cloned *ydaS* under the inducible P_BAD_ promoter, and *ydaT* under the P_T7_ promoter, in separate, compatible plasmids. Initially, we used *E. coli* strain BL21(DE3) and found that only *ydaT* expression caused a severe detrimental growth effect, and not expression of *ydaS*, as observed *via* cell samples spotted onto agar plates supplemented with appropriate inducers (Figure 8A). Further, inducing YdaS together with YdaT did not show any additive effect, so they are unlikely to be co-regulators. We also tested *ydaT* induction in strains MG1655 *rac*^+^ and *Δrac* (Figure 8B), and it was clear that the toxicity comes solely from YdaT activity, participating in an unknown pathway outside of the *rac* locus. Interestingly, *E. coli* B strain, ER2566, a derivative of BL21(DE3), showed complete loss of YdaT toxicity, indicating a possible lack of certain genetic mediators of filamentation (Figure 8B).

We also wanted to confirm that induction of *ydaT* or *ydaS* will not trigger Rac excision, so we repeated the Rac excision assay. Whether measuring cell growth or relevant qPCR amplification products (Figure 8C, D), both approaches showed that increased expression of *ydaS* induces Rac prophage excision (Figure 8D), but not *ydaT* (Figure 8C).

### Generation of Tn*5* library in Δ*rac* genetic context under *ydaT* overexpression

To find an effector gene for YdaT outside the *rac* locus, we again created a Tn*5* library, but this time in a Δ*rac* context. We used a plasmid carrying *ydaT* linked the P_BAD_ promoter, and then followed exactly the same protocol as before. We obtained a representative library in technical duplicates. This time, the screen of colonies on arabinose-LB agar plates did not reveal as many mutants with changed colony morphology as before. In fact, majority of regular colonies could not be passaged, having obvious viability problems. We managed to isolate just six Tn*5*-integration mutants. We identified three disrupted genes: three occurrences in *rcsA*, two in *cyaA*, and one in *pitA*. The *rcsA* and *cyaA* genes were among Tn*5* integrants determined before (Table 1, Figure 3). The *pitA* gene specifies low-affinity inorganic phosphate transport (Pit), and is the major uptake system for phosphate under conditions of P_i_ abundance (57). Of these three, we chose to examine the involvement of *rcsA* more closely.

### Toxic YdaT effect is alleviated when the *rcsA* gene is inactive

**Figure 9.**
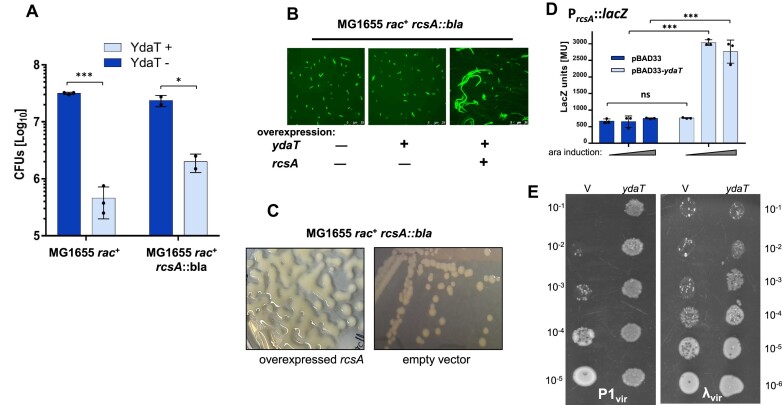
The RcsA transcription factor complements the toxic YdaT phenotype, suggesting their possible common interaction within the pathway leading to lipopolysaccharide biosynthesis. (**A**) Growth of MG1655*rac*^+^ cells when *ydaT* expression is low. The effect is alleviated when *rscA* is inactive (MG1655*rac*^+^*rcsA*::*bla*). The averages (± SD) are shown with Student's t-test: *P* *** *P*<0.0001; * *P*<0.05. (**B**) In contrast, only overexpression of two genes at the same time: *ydaT* and *rcsA* in *rcsA::bla* background restores the initial filamentous cell phenotype. (**C**) Overexpression of *rcsA* gene alone yields a mucoid, shiny colony morphology. (**D**) Miller assay showing increased *rcsA* promoter activity under *ydaT* induction. The error bars indicate the averages (± SD) of at least three independent measurements. Student's t-test was performed with values: *** *P*<0.0001; * *P*<0.05 and ns: *P*>0.05. (**E**) Bacteriophage test on cells under *ydaT* overexpression: P1_vir_ (left) and λ_vir_ (right). Serially diluted phages and the same amount of cells were spotted onto LB-agar plates. For λ_vir_ the efficiency of lysis is unchanged upon *ydaT* induction (in comparison to the empty vector (V) control). For P1_vir_ recognition receptor is a terminal glucose moiety of the lipopolysaccharide (LPS) for which synthesis is disturbed during *ydaT* induction.

We repeated the complementation assay for *rcsA* gene, which is a transcription factor affecting various bacterial pathways including cell division. We generated an *E. coli rac^+^ rcsA::bla* strain and transformed with two compatible plasmids, respectively carrying inducible *rcsA* and *ydaT* genes (Supplementary Table S1), with empty vectors as controls. The assay showed that MG1655 *rac*^+^ has 69-fold reduced viability when *ydaT* is overexpressed, but only 12-fold lower if the strain was *rcsA*-deficient (Figure 9A). We also confirmed that only combined overexpression of *rcsA* and *ydaT* genes in the *rcsA::bla* background could restore the altered phenotype from normal bacterial cells back to filamentous ones (Figure 9B). This is strong evidence that the RcsA TF plays a significant (though partial) role in the YdaT-initiated pathway leading to a cell division defect. In addition, we noticed that *E. coli* cells overproducing RcsA have a distinct colony morphology, with shiny, mucoid characteristics (Figure 9C).

To determine whether YdaT affects *rcsA* expression directly, we fused the *rcsA* gene (under its native promoter) to the *lacZ* reporter gene, and induced *ydaT* expression *in trans* (Figure 9D). Clearly, higher *ydaT* levels led to increased *rscA* expression, by almost five fold, indicating that YdaT is a gene regulator that activates *rcsA*, whether directly or indirectly. In addition, some reports have shown RcsA affects lipopolysacharide biosynthesis *via* interaction with RcsB (53). We wondered whether increased levels of RcsA/RcsB changes the LPS, using protection against phages that recognize LPS receptors as a biomarker for LPS alteration. P1_vir_ bacteriophage recognizes the LPS core oligosaccharide of *E. coli* K-12 as a receptor (58). λ_vir_ phage served as a negative control, as its LamB receptor protein should not be affected by LPS changes (59), though LPS alteration can reduce somewhat amounts of LamB on the cell surface (60). λ_vir_ is insensitive to repression by CI, so λ-lysogenic hosts are still susceptible. The assay, in which serially diluted phages and a constant amount of bacterial cells were spotted onto LB-agar plates, showed protection against P1_vir_ only in cells with elevated levels of YdaT (Figure 9E). The same cells were still comparable in plaquing efficiency when λ_vir_ phage was tested, irrespective of YdaT levels. Thus, it appears that the receptors recognized by phage are lost or changed when YdaT levels are elevated, as reflected by the loss of P1 phage efficiency of plaquing. More experiments are needed to verify whether P1 resistance is related to the change in the cell envelope or phage inability to reach its receptor.

## Discussion

### Cryptic Rac prophage uses a regulatory scheme similar to that of the Lambda phage immunity region

Our initial objective in this report was to find a function for YdaT protein, and assign the possible mechanism of its toxicity. However, our experimental approach led us to consider the broader phenomenon of Rac prophage maintenance versus excision. The Rac immunity region is located between *racR* and *ydaST*, and was previously investigated and noted to be similar to the immunity regions of other lambdoid prophages in terms of gene organization, operator locations, and gene regulation (10,16,24,26,61,62). Such regions typically impose the lysis *vs*. lysogeny decision. Our bioinformatic analyses have uncovered an intriguing discovery: while RacR and CI repressor exhibit low sequence similarity, they share a structurally similar N-terminal domain responsible for DNA binding. Remarkably, although the C-terminal domains of these proteins differ significantly in structure, they both serve as the foundation for oligomerization. Bioinformatics predictions, along with size-exclusion chromatography supports octamers in active form. Both repressors ensure that prophage DNA is maintained in the genome, by acting within the lysis/lysogeny decision region (between the CI and Cro genes in the case of bacteriophage λ) (63). RacR has multiple binding sites, comprising four inverted repeats between *racR* and *ydaS*, which cover the *racR* promoter (24). In this context, in Rac, the functional analog of the λCro protein (an antagonist of λCI) seems to be played by YdaS, as its increased expression leads to Rac excision, comparable to λCro protein, which switches λ phage from lysogeny into the lytic phase (64). However, unlike in active Lambda phage, Rac induction does not cause the cell lysis and plaques are not formed.

In a similar way, we also tested the proposal that YdaT acts as a λCII functional analog (61). In bacteriophage λ, CII activates several promoters during the switch to lytic growth (65,66). Under our conditions in *E. coli* MG1655, YdaT has no effect on Rac excision. Perhaps in this defective prophage excision is solely dependent on RacR concentration without any other TF involvement needed. Rac excision is not as rapid a process as λ phage excision (switch of cycle from lysogeny to lysis), which in λ occurs within a 2–3 h time window after Cro overexpression or CI proteolysis in rich media (67). In our case, Rac excision (under RacR deficit), takes generations, not hours, and never ends with cell lysis. In addition, interestingly, the YdaT-like protein of the lambdoid prophage CP-933P does not have any toxic effects (61), whereas Rac YdaT yields serious viability problems, depending on the host genetic context (Figure 8). Thus again, Rac prophage regulation and the gene functions it encodes are distinct from other Rac-like regions described so far, and require further study to reveal its peculiarities.

### Rac excision and increased YdaT levels are coupled

The RacR regulon has not yet been characterized in detail, and there are several genes without determined functions, such as the *racR*-proximal operons *ydaFG* and *ydaUVW* (Figure 1B). The RacR regulon was previously reported as being essential to *E. coli* (26,27,68). Due to variation in Rac genes content across *E. coli* strains (26), the RacR regulon might also vary. Quite often, gene manipulation within the RacR regulon triggers a lethal effect manifested by growth defects (26,55,69,70). The Keio collection of *E. coli* single gene deletion mutants does not contain a Δ*racR* mutant (71), consistent with our observation that such mutation is possible only in Δ*ydaST* background. The gene for YdaT is completely silenced under normal conditions by RacR repressor (24,26). Previously, YdaS/YdaT action was linked indirectly to inhibition of cell division by acting on DNA replication or chromosome segregation (72). YdaT is not only a TF acting *in cis* within Rac (work in progress), but also has target sites outside of the Rac region. Thus, YdaT-mediated toxicity may be a diverse process, affecting several target genes. Recently, a DNA target site for the YdaT ortholog from the *E. coli* CP-933P lambdoid prophage was determined, which may help to identify other putative genomic sites of YdaT interaction (73). No toxic effect has been shown for this ortholog, but there is only ∼30% amino acid identity between the YdaT orthologs from Rac and CP-933P, thus their functions might differ to some extent.

Our transcriptomic data revealed that, under decreased RacR expression, expression of certain Rac genes increases substantially (23). This should help to identify members of the RacR regulon, as well as other gene associations (24). The global regulator H-NS can also trigger Rac prophage excision (74). Nevertheless, under conditions of RacR deficit, caused in this study by C protein action, two processes are initiated: (i) RacR regulon upregulation, including YdaT and (ii) Rac excision due to rise of *xisR* and *intR* expression. We hypothesize that these two pathways, although functionally independent, are coupled. As YdaT toxic activity accumulates in cells (possible routes described below), it seems that Rac excision occurs in parallel. The Rac prophage is defective for replication, so its circular DNA might be maintained in cells for several generations and subsequently be lost, which also possibly is what halts Rac gene expression.

Overall, we propose that the Rac excision acts as a countermeasure to cell lethality due to YdaT action. Cells can apparently survive the relatively short ‘toxic’ period, until they are cured of Rac prophage. We previously observed that the filamentation process is reversible with long-term culturing (23), but now we believe that the cells either die or lose the Rac DNA to survive. This may represent an excellent example of how bacteria use different regulatory strategies involving master regulators (here RacR) to maintain beneficial prophage genes, while silencing those likely to be deleterious. Non-cryptic (intact) prophages are likely to kill the cell upon induction of the lytic cycle, so there should be a strong evolutionary selection for mutations leading to inactivation of prophage lytic capabilities (14,75).

### YdaT-dependent deficiencies in lipopolysaccharide biosynthesis and cell division

**Figure 10.**
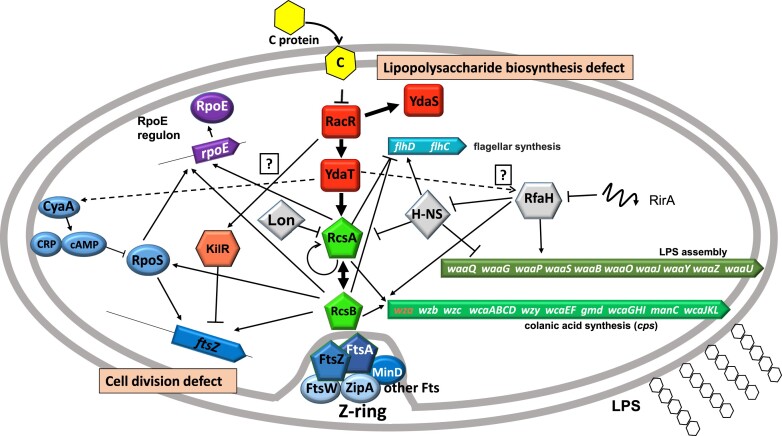
Hypothetical model for a regulatory TF’s cascade triggered by C protein and resulting in YdaT-dependent deficient lipopolysaccharide biosynthesis and cell division. C protein (yellow hexagon) is accepted as a foreign TF, which affects a Rac locus TFs (red blocks): RacR, YdaS, YdaT. In turn, YdaT affects a genomic TF, an RcsA TF, which together with RcsB (green blocs) modulate exopolysaccharide biosynthesis. Thick lines indicate the experimentally verified pathway explained in this work, normal lines pathways known from the literature data and dashed lines pathways to be clarified in the future. Description explained in Discussion.

We confirmed that foreign TF entry into a cell (C.Csp231I in this case) may perturb existing regulatory networks in a chain reaction. We showed previously that C protein directly inhibits RacR repression of its regulon (23,24), which boosts expression of, in particular, two more TFs: YdaS and YdaT. Here we tried to define the further steps in the molecular mechanisms of the toxic effect initiated by YdaT. We used Tn*5* library analysis, transcriptomic data, and other reports (Figure 10, Supplementary Figures S9, S10). Some missing links were proposed and will be tested in the future. Unexpectedly, it seems YdaT may not only act *in cis*, in its gene's upstream region, but also at other effector sites in the *E. coli* genome. The transposon mutagenesis screening identified at least one effector gene, again a TF, RcsA, of the two-component regulatory Rcs system. The Rcs system can sense cell envelope damage or defects, and regulate gene expression to counteract this stress (53). In particular, RcsA alone is an autoactivator (76), but forms a heterodimer with another partner TF, RcsB, that can activate not only the *rcsA* gene, but other genes as well, including the *wza* gene of the exopolysaccharide and colanic acid biosynthesis (*cps*) operon in enteric bacteria (77,78) (Figure 10). This is in accord with our observation that some mutants have mucoid colonies, as is seen in *rcsA* overexpression (Figure 9C) or in *lon****^−^*** cells (RcsA proteolysis absent) (54,79). Expression of *cps* operon genes is also upregulated in our C.Csp231I protein-producing cells (Supplementary Figure S10), supporting our hypothesis that YdaT might mediate a regulatory cascade leading from C protein to the *cps* operon (Figure 10). A gene involved in colanic acid synthesis (*wcaD*) was also detected in our library (Table 1, Supplementary Figure S9). The genetic complexity gets higher if we add global stress regulators to the model, such as the sigma factor RpoE, which initiates regulon expression in response to extracytoplasmic stress (80). The *rpoE* promoter is positively regulated by RcsAB in response to defects in LPS core biosynthesis (81). *E. coli* in conditions of cell envelope stress or morphology defects cannot regenerate its normal rod-shape unless RcsB activity is present (82). In addition, RcsAB represses motility *via* negative control of *flhDC* expression (83,84), which is also observed in our transcriptomic data (strongly decreased expression of *flhD* and *flhC* genes, Supplementary Figure S10). We clearly have shown a positive effect of YdaT on the promoter activity of *rcsA*, whether direct or indirect, and more detailed work is in progress (Figure 9D).

We noticed in our C protein – responsive Tn*5* library a large representation of genes involved in LPS biosynthesis (six insertion within the *waa* operon and RirA regulatory RNA, Table 1). In our transcriptomic analysis with C-expressed cells, at least 16 genes involved in LPS biosynthesis exhibited reduced expression, including *wbbI*, *wbbJ* and *wbbK* (Supplementary Figure S10). Hence we infer that YdaT expression significantly changes a lipopolysaccharide composition. This is consistent with our observation that YdaT-expressing cells were resistant to P1_vir_ phage, for which the terminal glucose moiety of the lipopolysaccharide is the recognition receptor (Figure 9E). In addition, production of mucoid layer by stimulation of *cps* operon might mask some phage receptors, thus preventing the cell against phage lysis. Cell conversion to becoming phage resistant is usually associated with losing a part of the outer core sugars, having certain modifications in the LPS or masking a surface phage receptors (59,85), usually as an effective tool in the bacteria arms race (86–88). Although, Rac prophage being cryptic cannot fully compete with other active phages, still some remaining genes, like *ydaT* might be also considered as a putative functional superinfection exclusion system helping Rac to avoid its host lysis by other active phages. More addressed approaches are needed to verify this highly speculative hypothesis.

We also hypothesize that YdaT may indirectly inhibit the expression of transcription elongation factor *rfaH* (Figure 10). RfaH is a specialized antiterminator, required for expression of genes that encode LPS core, capsule biosynthesis enzymes, toxin-antitoxin systems and some dedicated secretion systems in *Enterobacteriaceae* (89). The small regulatory RNA, RirA binds to RfaH to inhibit its effect so that LPS assembly (by *waa* operon) is expressed in a balanced manner (81,90). The regulatory links between RfaH and RcsA may involve the global regulator H-NS (Figure 10). Indeed, in our transcriptome data, expression of *hns* was decreased almost five-fold in C protein-expressing cells, and a Tn*5* insertion (isolated in the presence of Rac) was found within the *rirA* regulatory RNA gene (Table 1). H-NS functions almost exclusively as a transcriptional repressor, and its activity may be opposed by RfaH, so they may function as silencer/ countersilencer pair (91). In cells lacking *hns*, pathways leading to LPS and cell envelope synthesis were overexpressed (92). In turn, H-NS and Lon together have the highest negative effect on RcsA, H-NS by inhibiting the *rcsA* promoter, and Lon by proteolysis of RcsA itself (54). Thus, it seems the effect of YdaT on *rcsA* expression might also be mediated by H-NS, RfaH and RirA (Figure 10). This needs to be verified experimentally.

In parallel, RcsAB may modulate cell division by directly activating the *ftsZ* promoter. FtsZ gene expression depends on several upstream promoters (93), and FtsZ is an essential cell division protein responsible for septum formation (94). FtsZ polymerizes into a dynamic ring (Z-ring) that defines the division spot and recruits other proteins to drive localized peptidoglycan synthesis (95). We observed in C protein/YdaT-expressing cells, a defect in Z-ring formation within cell filaments (Figure 2A). The Rac protein, KilR is a potent inhibitor of FtsZ (96), but in our case the same filaments are formed in the absence of *kilR* (23).

Our schematic model of the cross-talking TF cascade in *E. coli* (Figure 10) shows some triggered pathways that might be verified experimentally. It is also challenging, amid this complexity, to separate the primary genetic signal from the secondary outputs or feedbacks. Of note, YdaT might stand as an interesting prophage-encoded example of a TF with pleiotropic effects on *E. coli* physiology. Another prophage-origin TF, AppY, was identified that triggered regulatory pathways increasing bacterial survival under low pH conditions, and also causing biofilm formation and decreased motility (97). Both studies provide molecular insights into prophage-encoded TF integration into the *E. coli* regulatory network. The comprehensive picture of bacterial physiology is inextricably linked with prophages, with benefits for both parties (98). Phage integration into a bacterial chromosome is highly-effective way to be replicated along with the rest of the bacterial genome (99). Bacteria can benefit in many ways including: phage immunity, antibiotic resistance, virulence factors, motility, new nutrient metabolism, biofilm formation, and overall a high level of heterogeneity (13,55,74,100–104). These collectively contribute to more efficient and competitive behavior of bacteria to thrive in various microhabitats and ecological niches. However, as shown here, prophages can also complicate horizontal gene transfer by interfering with existing regulatory networks.

## Supplementary Material

gkad1234_Supplemental_File

## Data Availability

No new data were generated or analysed in support of this research. The RNA-seq data (23) are available in the NCBI GEO (accession number GSE126248). The data from the bioinformatics analyses are available through the Repository for Open Data (RePOD) at https://doi.org/10.18150/FV9R0P.
