# Supplementary material for: Lethal perturbation of an Escherichia coli regulatory network is triggered by a restriction-modification system's regulator and can be mitigated by excision of the cryptic prophage Rac
Source: Nucleic Acids Res. 2023 Dec 28;52(6):2942–60. doi: 10.1093/nar/gkad1234 (PMC11014345; doi:10.1093/nar/gkad1234)
Supplement: gkad1234_Supplemental_File [file gkad1234_supplemental_file.pdf]

Supplementary Material for:

Lethal perturbation of an *Escherichia coli* regulatory network is triggered by a restriction-modification system's regulator and can be mitigated by excision of the cryptic prophage Rac

Running title: Exogenous transcription factor depresses RacR repressor level, causing Rac excision and causing toxic expression of YdaT.

Key words: transcription regulation / transcription factor / promoter / operator / DNA-protein interaction / DNA binding / transcriptional cross-talk /

Katarzyna Gucwa[1], Ewa Wons[1], Aleksandra Wisniewska[1], Marcin Jakalski[2], Zuzanna Dubiak[1], Lukasz Pawel Kozlowski[3] and Iwona Mruk[1]*

1 Department of Microbiology, Faculty of Biology, University of Gdansk, Wita Stwosza 59, Gdansk 80-308, Poland

2 3P-Medicine Laboratory, Medical University of Gdansk, Debinki 7, 80-211, Gdansk, Poland

3 Institute of Informatics, Faculty of Mathematics, Informatics and Mechanics, University of Warsaw, 02-097 Warsaw, Poland

*Corresponding author

Abbreviations: *E. coli*, *Escherichia coli*; TF, transcription factor;

Address for correspondence:
Dr. Iwona Mruk
Department of Microbiology, University of Gdansk,
Wita Stwosza 59, 80-308 Gdansk, Poland
Tel: (+48) 58 5236071
e-mail: shamrock127@hotmail.com

Strains, plasmids and oligonucleotides

Bacterial strains and plasmids

The bacterial strains and plasmids used in this study are listed in Table S1, oligonucleotides in Table S2.

Table S1. *Escherichia coli* strains and plasmids used in the study

Strain or plasmid	Description	Source
MG1655	K-12 F- λ- *ilvG- rfb-50 rph-1*	(1)
MG1655 rcsA::bla	*rcsA* inactivated by *bla* gene, Amp^R	this work
BW25113	K-12; F- λ- *$lacI^q$ rrnB3* Δ*lacZ4787 hsdR514* Δ*(araBAD)567* Δ*(rhaBAD)568 rph-1*	(2)
BW25113 Δrac	as BW25113, but deletion of entire rac locus (23060 bp)	(3)
MG1655 Δrac	as MG1655, but deletion of entire rac locus (23060 bp)	(4)
MG1655 ralRA::bla	*ralA* replaced with *bla* gene, Amp^R	(4)
MG1655 ydaS::bla	*ydaS* inactivated by *bla* gene Amp^R	(4)
MG1655 ydaT::bla	*ydaT* inactivated by *bla* gene, Amp^R	(4)
BW20767	RP4-2-Tc::Mu-1 *kan*::Tn*7* integrant, *leu-63*::IS*10* *recA1 zbf-5 creB510 hsdR17 endA1 thi* *uidA*(ΔMluI)::*pir*+<br>Host of pRL27, Kan^R	(5)
MC1061	*araD139* Δ(*ara, leu*)7697, Δ*lacX74*, *galU*, *galK*, *hsdR*, *strA*	(6)
DH5α	F- λ- *recA1 endA1 gyrA96 thi-1 hsdR17 supE44 relA1 deoR(lacZYA-argF)U169*	
DH5α λpir+	as DH5α but λ*pir*+<br>Host of pRL27	(7)
BL21(DE3)	B $F^–$ *ompT gal dcm lon* $hsdS_B(r_B^- m_B^-)$ λ(DE3 [*lacI lacUV5-T7p07 ind1 sam7 nin5*]) $[malB^+]_{K-12}(\lambda^S)$	Novagene
ER2566	B F– λ– *fhuA2 [lon] ompT lacZ*::T7.1 *gal sulA11* Δ(*mcrC-mrr*)114::IS10 R(*mcr-73::miniTn10*)(TetS)2 R(*zgb-210*::Tn10)(TetS) *endA1* [dcm]	(8,9)
pRL27	mini-Tn*5* transposon (*oriR*6K) delivery vector, conjugative plasmid, Km^R,	(10)
pBAD33	vector with arabinose inducible araBAD promoter (P_{BAD}), *araC* repressor, pACYC ori, Cm^R	(11)
pBAD-CWT	for inducible expressing C WT protein	(12)
pBAD-Cmut	as pBAD-CWT, but C gene mutated, unable to bind DNA	(12)
pBAD-racR	as pBAD33, but *racR* gene under inducible P_{BAD} promoter	(4)
pHM1786 (pHM1501)	for cloning of genes identified by Tn*5* mutagenesis, a pGB2 derivative (*ori* pSC101, IPTG inducible P_{tac} promoter, Spc^R)	(13,14)
p51-ydaT	pLATE51 deriviate (Thermo Scientific) plasmid carrying *ydaT* gene with N-terminal His-tag under P_{lacUV5} promoter, induced by IPTG; pMB1 replicon.	this work

Strain or plasmid	Description	Source
	ydaT gene was amplified by PCR (primers ydaT51F and ydaT11R) and annealed with pLATE51 with aLICator LIC Cloning & Expression System (Thermo Scientific)	
pBAD-ydaT	as pBAD33, but *ydaT* gene under inducible P_{BAD} promoter. Plasmid was constructed via Gibson assembly with *ydaT* gene amplified by PCR (primers ydaTpBAD33Fwd and ydaTpBAD33Rev), and vector pBAD33 with primers pBAD33ydaTFwd and pBAD33ydaTRev	this work
pBAD-ydaS	as pBAD33, but *ydaS* gene under arabinose inducible promoter, CmR	(15)
pLexRcsA::lacZ	as pLEX3B, but carrying the upstream sequence with P_{rcsA} and fragment of *rcsA* coding sequence (from -390 to +348 in reference to the +1 translation initiation site of *rcsA*). The fragment was inserted leading to creation of the genetic fusion in frame *rcsA::lacZ.* Plasmid was constructed via Gibson assembly with up-stream sequence and fragment of *rcsA* gene amplified by PCR (primers rcsApLexFwd and rcsApLexRev), and vector pLex3B with primers pLexrcsAFwd and pLexrcsArev	this work
pDSW360	*ftsW* gene in *gfp* fusion (FtsW::GFP) under P_{tac} promoter, to localize the cell division spot, pBR322 ori, kanR	(16)
p24	pBR322-derivative plasmid carrying the genes of Csp231I RM system with REase gene inactive (MTase, C protein WT)	(12)

Table S2. The oligonucleotides used in this study.

**Experiment**	**Primer**	**Sequence (5'→3')**
PCR Rac prophage	intR for	GGGGCATGAAACTGCGCAGATGG
	ynaE rev	CCACTTTAGGGTGTCTTTTGATTTC
	ttcA rev	GCCCGGTTGCTTTTGATCGAGG
	ydaSpLEX3B for	ATTACTCGAGCGACGTTCGGCATGTTGTTCGC
	ydaTpLEX3B rev	ATTGCTCGAGGCATTGGCTACCCATGCCTGCC
	PRracRpLEX3	CCTAAAGCTTTTTTGTATCTGAATTGGTGG
	PFracRpLEX1	AAATCTCGAGTTGCTTGAATGAATAGTTCTC
	ydaS for	CTATTCATTCAAGCAAGCTTGTGC
	ydaS rev	CTGTCATTTAGTTGCTTCACTGAC
	ydaT for	CAAGCATGAGCACATCGAATC
	ydaT rev	GAACAATGACAGAATCGTCGG
qPCR	idnT rev	CCCTCCTTCTGGAACAGTATTT
	idnT for	TCGATGGTGCGTCCATTAC
	zntB qPCR for	CGCAACGTGATGTTTATGCTC
	zntB qPCR rev	CTATACAGGCGTCGATTTCG
	intR qPCR	CGGTTATCGTAGTTGCAAAG
	ttcA qPCR	GTCTTTACCCCCGGAGAG
Recombineering	ttcA bla for	CTTCAATCATATTGAAGTCAGCAATGGCTTCGCCCACGTTACGACGCAGACGCATTCAAATATGTATCCGCTC
	intR bla for	ATTTCAGTTCTCTGGTACTAAATGGGGCAAATTGGGGGCAAACTTTGCAACATTCAAATATGTATCCGCTC
	xisR bla rev	CATCGGGCGTCAATTTCAGTCTAACATTGGCGCCTGCCAGAGGTGATGCGTTACCAATGCTTAATCAGTG
	waaQ bla for	GATGCGATATCATGGGGATATGTTATTAACTACTCCTGTCATCAGTACGCTCATTCAAATATGTATCCGCT
	waaQ bla rev	CGATCATAATTGTGCATTCCTGTCAGCTGAAGGGGCATCTTCTGGCAACAGCTTTACCAATGCTTAATCAGTG
	rcsA bla for	GCCATGTCAACGATTATTATGGATTTATGTAGTTACACCCGACTAGGTTTAACCATTCAAATATGTATCCGCT
	rcsA bla rev	GTTAGCGCATGTTGACAAAAATACCATTAGTCACATTATCCGGGTCTGACAGTTACCAATGCTTAATCAGTG
	nlpA bla for	ATGAAACTGACAACACATCATCTACGGACAGGGGCCGCATTATTGCTGGCCATTCAAATATGTATCCGCT
	nlpA bla rev	GAATTACCAGCCAGGCACCGCGCCACCGTTAAAAATGGTTTCTGCCGCTTTAGTTACCAATGCTTAATCAGTG
	bla diagn for	GTAACTCGCCTTGATCGTTG

Experiment	Primer	Sequence (5'→3’)
	xisR bla diagn rev	CAGTCCTCGAGAATTGCATTG
	waaQ diagn rev	GATGCAATGCGCATAAAGTC
	rcsA diagn rev	CACAATGTACCTGGTTTCGC
	nlpA diagn rev	GCTGTGCAGAACCATTAAGTAGC
Sequencing	lacBSu	CGTAACCGTGCATCTGCCAGTTTG
	pBAD for	GCTATGCCATAGCATTTTTATCC
	pHM1786 rev	GGAGATATATCATGAAAGGCTGGC
Complementation	arnB for	GGCCGCGAATTCCCTTAAAATTTCGCTCCAAATGGC
	arnB rev	GCGCGCAAGCTTGCTTTCCTGCTCGTTATAAACGGG
	fadE for	GGAATCGAATTCCAAGTGGTCAGACCTCCTAC
	fadE rev	AAGGCTAAGCTTTTACGCGGCTTCAACTTTC
	nlpA for	GAGTCGAGAATTCGCAGCGACCTTACCGCTATAG
	nlpA rev	GATTTAAAGCTTCGTCCGGCGCGGCATAAGTG
	phoE for	AAGTCAGAATTCCGCGTTAATTAAAATCAGG
	phoE rev	TGAACTAAGCTTTTAAAACTGATACGTCATGC
	rcsA for	CACACAGAATTCGTAAGGGGAATTATCGTTACGC
	rcsA rev	CACACAAAGCTTCAGAATGTGTTAGCGCATG
	wcaD for	GCATCTGAATTCCAGTGGACAACAGATGCTGGAGG
	wcaD rev	GGCCGCAAGCTTCTTCGAGGTTACGAAACGCGAC
	yage for	GATCAAGAATTCGAACAAAATGATTCAGCAAGGAGATCTC
	yage rev	AAGGTCAAGCTTTCAGCAAAGCTTGAGCTGTTG
	yagK for	GGCCGCGAATTCGACCGTAGGACCAAATACAGG
	yagK rev	GGCCGCAAGCTTCTCACTGAGAAGGCCATTACC
Cloning	ydaT51F	GGTGATGATGATGACAAGAAAATCAAGCATGAGCAC
	ydaT11R	GGAGATGGGAAGTCATTAATGAACAATGACAGAATCG
	ydaTpBAD33Fwd	GGAATTCACCATGAAAATCAAGCATGAGC
	ydaTpBAD33Rev	GATTATAGTTTTAATGAACAATGACAGAATCG
	pBAD33ydaTFwd	TGTTCATTAAAACTATAATCTAGAGTCGACC

Experiment	Primer	Sequence (5'→3’)
	pBAD33ydaTRev	TGATTTTCATGGTGAATTCCTCCTGCTAG
	rcsApLexFwd	GATCCCTCGATGGCGGCATTAATGCCGTTAATTG
	rcsApLexRev	CGGGAGCAAGGATATCGTCGAGAGATTCCGG
	pLexRcsAFwd	CGACGATATCCTTGCTCCCGTCGTTTTAC
	pLexRcsARev	AATGCCGCCATCGAGGGATCTTCCTATG
Tn5 mutagenesis	tpnRL17-1	AACAAGCCAGGGATGTAACG
	tpnRL13-2	CAGCAACACCTTCTTCACGA

Table S3. PCR conditions I for amplification of transposon Tn*5* flanking regions.

Step	Phase	Temp. [°C]	Time [s]
1	Pre-denaturation	95	30
2	Denaturation	95	30
3	Annealing	74 - 66	15
4	Extension	72	60
5	Denaturation	95	30
6	Annealing	64 -56	15
7	Extension	72	60
8	Final extension	72	300

Note: Steps 2 - 4 repeated 12 times (4 degrees reduction of annealing temperature every 4 cycles). Steps 5 - 7 repeated 20 times (2 degrees reduction of annealing temperature every 4 cycles)

Table S4. PCR conditions II for amplification of transposon Tn*5* flanking regions.

Step	Phase	Temp. [°C]	Time [s]
1	Pre-denaturation	95	60
2	Denaturation	95	30
3	Annealing	66 - 46	15
4	Extension	72	200
5	Final extension	72	300

Note: Steps 2 - 4 repeated 44 times (2 degrees reduction of annealing temperature every 4 cycles)

Table S5. Identification of Tn*5* transposon insertion sites.

& Locus according to the sequence deposited with NCBI GenBank number U00096.3

$ Positioning according to the *Escherichia coli* K-12 substr. MG1655, version 26.5 on the website www.ecocyc.org corvengent with U00096.3

# The first and last nucleotide position of the given gene. Positioning as above.

* cells carrying plasmids p30 vs p18*; if the cell is empty log_2 fold change was between -1 and 1, so insignificant.

Tn5 mutant number	Gene or place of Tn5 insertion	Gene product	Length (bp)	locus tag &	Localization of the Tn5 $	Map position #	Rac presence	RNA-seq (fold-change) * (C- vs. C+)
35	*kefC*	glutathione-regulated potassium-efflux system protein KefC	1863	b0047	47769	47,769->49,631	–	
93	*thiP*	fused thiamin transporter subunits of ABC superfamily: membrane components	1611	b0067	72911	72,911<-74,521	–	1,08
37	*yadN*	putative fimbrial-like adhesin protein YadN	585	b0141	156299	156,299 <- 156,883	–	
27C/27B	*yaeF*	peptidase C92 family protein YaeF	825	b0193	216233	216,179 <- 217,003	+	
20	*fadE*	acyl-CoA dehydrogenase	2445	b0221	242 765	240,859 <- 243,303	+	
31	*phoE*	outer membrane porin PhoE	1056	b0241	259 990	259,045 <- 260,100	+++	
77	*yagE*	CP4-6 prophage; putative 2-dehydro-3-deoxygluconate aldolase	909	b0268	282 300	282,278 -> 283,186	–	
54	operon yagK	operon yagK, CP4-6 prophage		b0277	293 075	ok 292,500 -> 293,200	–	
105	*rclB*	DUF1471 domain-containing protein RclB	237	b0303	318331	318,331 <- 318,567	–	
49	*yahN*	amino acid exporter for proline, lysine, glutamate, homoserine	672	b0328	345666	345,666 <- 346,337	–	-1,38
80	*ybaK*	Cys-tRNAPro and Cys-tRNACys deacylase	480	b0481	506 881	506,603 <- 507,082	+++	
34	*ybdL*	methionine transaminase	1161	b0600	633 678	633,586 -> 634,746	–	
104	*ycgL*	PF05166 family peptide YcgL	327	b1179	1 227 801	1,227,681 -> 1,228,007	–	-1,01
57	*dgcJ*	putative diguanylate cyclase	1491	b1786	1872041	1,872,041 -> 1,873,531	–	
8	*rcsA*	DNA-binding transcriptional activator, co-regulator with RcsB	624	b1951	2023968	2,023,968 -> 2,024,591	+	
48 / 108	*wcaD*	putative colanic acid polymerase	1218	b2056	2 130 451	2,129,661 <- 2,130,878	–	1,19
36	*arnB*	UDP-4-amino-4-deoxy-L-arabinose aminotransferase	1158	b2253	2 366 760	2,365,910 -> 2,367,067	–	
40	*pdeA*	putative c-di-GMP phosphodiesterase PdeA	2190	b2395	2 516 100	2,515,643 <- 2,517,832	+	
55	*csdE*	sulfur acceptor protein CsdE	444	b2811	2 944 849	2,944,542 -> 2,944,985	+++	
68	*yqeG*	putative transporter YqeG	1230	b2845	2 986 980	2,985,847 -> 2,987,076	–	-1,36
97	*yqeL*	uncharacterized protein YqeL	81	b4683	2989819	2,989,819 <- 2,989,899	–	
42	*aaeR*	LysR-type transcriptional regulator AaeR	930	b3243	3 389 668	3,389,520 -> 3,390,449	–	

103	*yiaL*	DUF386 domain-containing protein YiaL	468	b3576	3743743	3,743,743 -> 3,744,210	–	-1,13
46	*waaH*	UDP-glucuronate:LPS(HepIII) glycosyltransferase	1035	b3615	3789047	3,789,047 <- 3,790,081	–	
29	*waaZ*	lipopolysaccharide core biosynthesis protein WaaZ	852	b3624	3800147	3,799,345 <- 3,800,196	–	
61	*waaG*	lipopolysaccharide glucosyltransferase	1125	b3631	3 806 442	3,805,943 <- 3,807,067	+++	
67	*waaQ*	lipopolysaccharide core heptosyltransferase 3	1035	b3632	3 807 472	3,807,064 <- 3,808,098	–	
63	*waaQ*	lipopolysaccharide core heptosyltransferase 4	1035	b3632	3 807 482	3,807,064 <- 3,808,098	–	
47	*rirA*	small regulatory RNA RirA (RfaH interacting RNA)	73	b4760	3 808 201	3,808,166 <- 3,808,238	+	
81	*nlpA*	lipoprotein-28	819	b3661	3 839 916	3,839,175<-3,839,993	+	-4,46
4	*rbsK*	ribokinase	930	b3752	3937294	3,937,294 -> 3,938,223	–	-2,19
83 / 94	*cyaA*	adenylate cyclase	2547	b3806	3 991 182	3,991,153 -> 3,993,699	+++	
78 / 17	*arpA*	regulator of acetyl CoA synthetase	2187	b4017	3937294	4,220,301 <- 4,222,487	+++	-2,08
18	*phnC*	phosphonate/phosphate ABC transporter ATP binding subunit	789	b4106	4 324 426	4,324,377 <- 4,325,165	+++	
53	*idnD*	L-idonate 5-dehydrogenase, NAD-binding	1032	b4267	4493375	4,493,375 <- 4,494,406	–	-1,56
91	*yjhI*	KpLE2 phage-like element; putative DNA-binding transcriptional regulator YjhI	789	b4299	4 525 231	4,525,015 <- 4,525,803	+++	
100	*yjhZ*	putative acetyltransferase YjhZ, KpLE2 phage-like element	246	b4657	4 534 545	4,534,430 <- 4,534,675	+	
84	*idlP*	iraD leader peptide	84	b4722	4 556 938	4,556,913 -> 4,556,996	–	

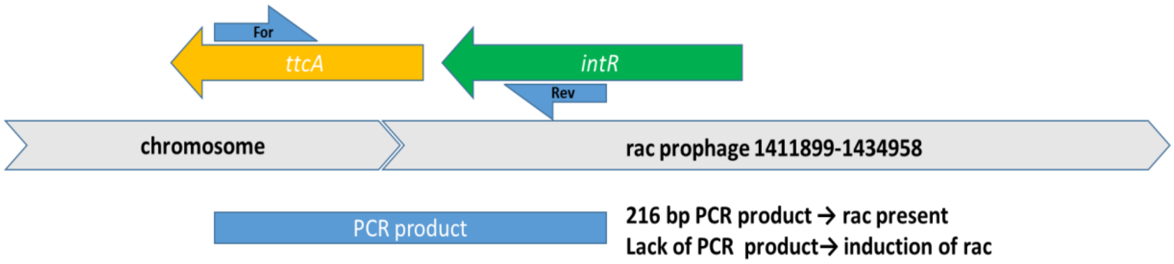

Figure S1 Schematic location of primers used to evaluate the phage presence or absence (phage induction) by q-PCR.

Transposon insertion localization

Inverse PCR analysis and electrophoresis confirmed random Tn5 transposon insertion (Table S5). BLAST analysis of the obtained sequences let the identification of Tn5 integrants. Representative PCR products obtained for majority of integrants.

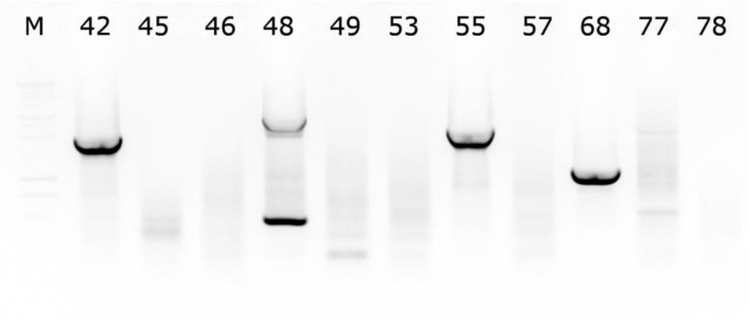

Figure S2. Random insertion of Tn5 transposon in the MG1655 *rac*$^{+}$ genomic DNA. Results obtained by inverse PCR. The numbers indicate the designations of mutants, M stands for 1 kb DNA ladder. Individual digestion and amplification conditions were needed for different Tn*5* mutants to obtain a positive PCR product.

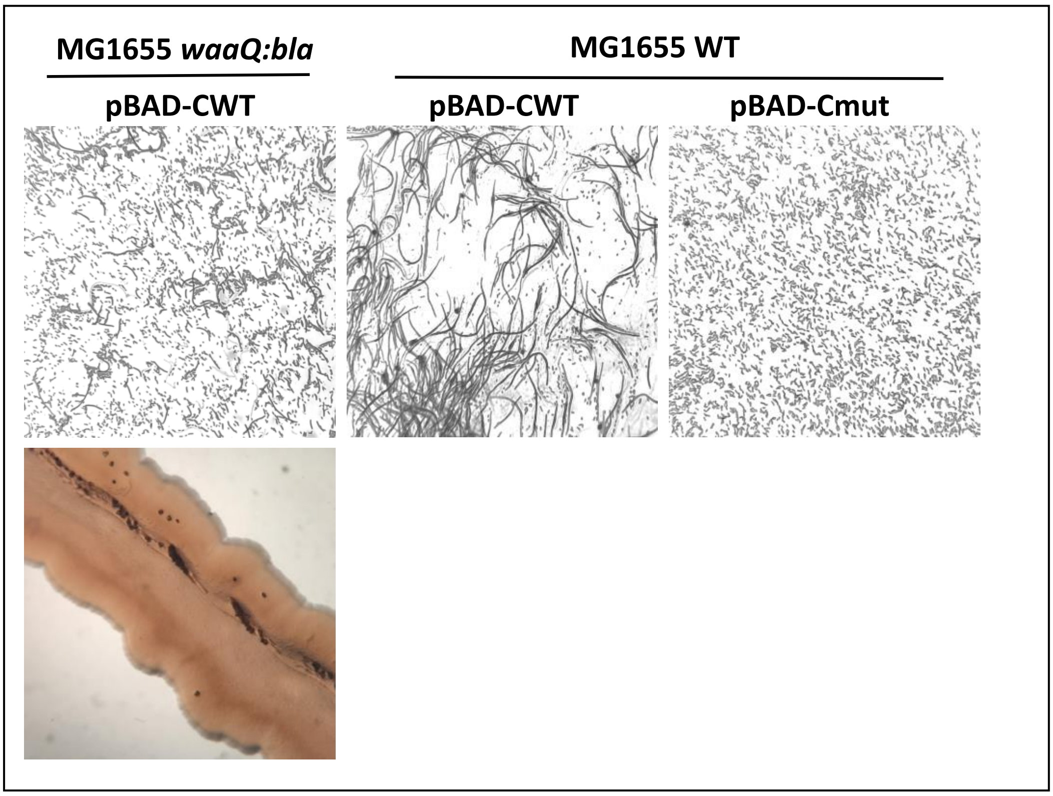

Figure S3. Directed mutagenesis to interrupt a *waaQ* gene with *bla* cassette resulted in cell filamentation loss in C protein presence.
Upper panel, morphology of MG1655 WT cells after induction of C gene (CWT, control) expression (0.1% ara on LB-agar plates) or its mutated version (Cmut).
Lower panel - lack of curly phenotype in MG1655 *waaQ:bla* deletant mutant strain with C gene expression. The *waaQ* Tn*5* mutant was *racR* positive in PCR (Rac prophage still present in the genome).

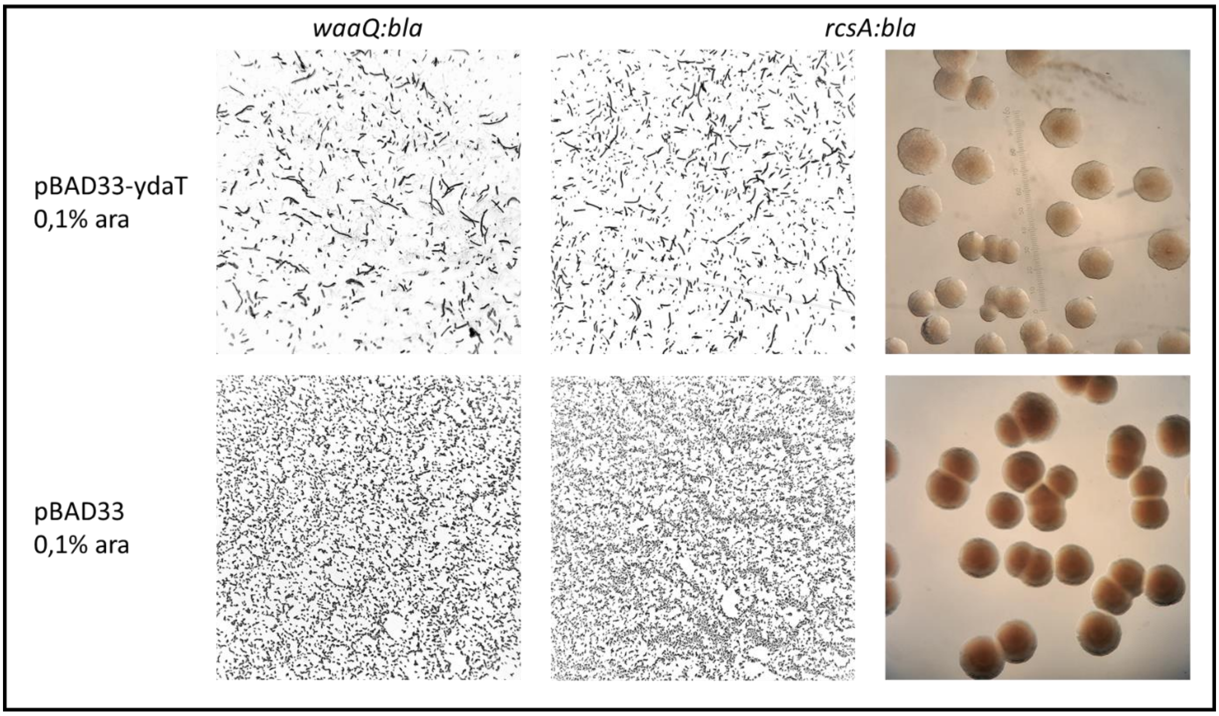

Figure S4. Directed mutagenesis to interrupt the *waaQ* and *rcsA* genes with *bla* cassette resulted in cell filamentation loss after YdaT expression induction.
Cell morphology of MG1655 *waaQ::bla* or *rcsA::bla* cells after induction of *ydaT* wild type gene (pBAD33-*ydaT* and pBAD33 as a negative control). For *rcsA::bla* mutant, colonies are also shown.

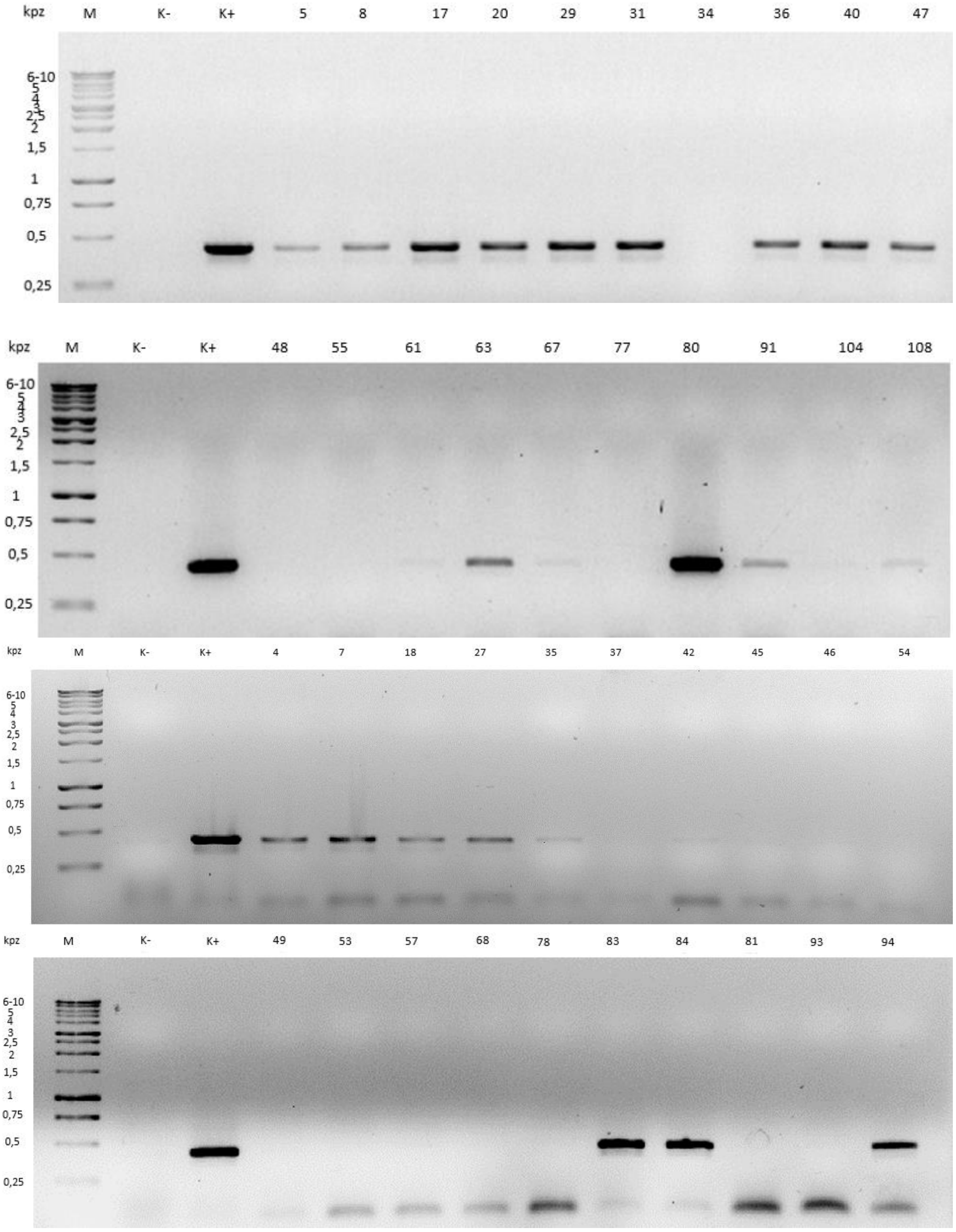

Figure S5 Determination of Rac presence in the library mutants. The 400 bp product was amplified with PCR Rac prophage primers: PRracRpLEX3 and PFracRpLEX1. The samples were run on 1% agarose gel at 100 V, stained with ethidium bromide. The numbers indicate the mutant, as in Table 1 of main manuscript or Table S5 of supplement, M stands for 1 kb DNA ladder, K+ stands for positive control *E. coli* MG1655*rac*$^{+}$, K- stands for negative control *E. coli* MG1655Δ*rac*.

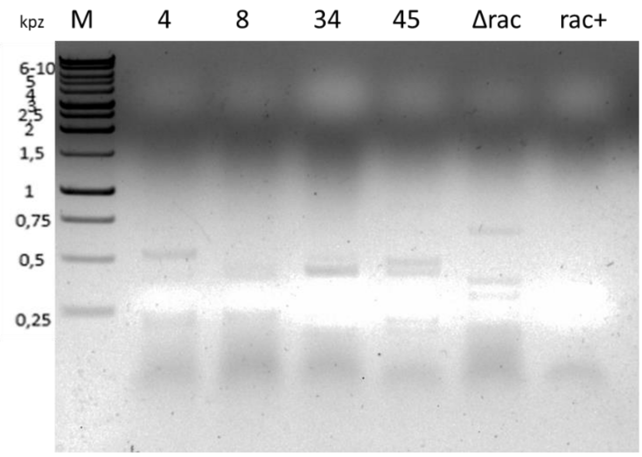

Figure S6. Determination of Rac circle presence after Rac excision. The 962 bp product was amplified with *intR* and *ynaE* primers - the first and the last gene of 23 kbp Rac prophage. In normal situation (Rac in the genome) they are too far to create PCR product, however after prophage induction and forming a circle, this two genes would stand next to each other allowing amplification of 962 bp product if the circle would be still present. Lack of the product with the equal absence of 400 bp *racR* product (as in Fig S5) indicates circle loss.

The files related to the bioinformatics protein modelling are available at RePOD repository at https://doi.org/10.18150/FV9R0P

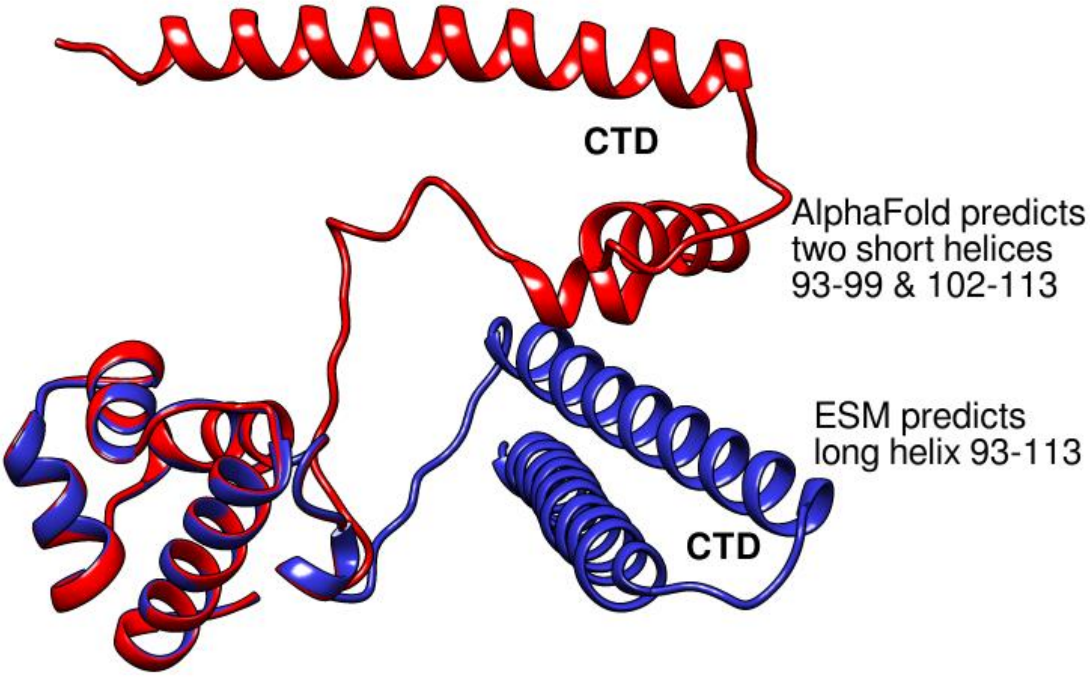

Figure S7. Difference in RacR modelling. N-terminal domain (left) responsible for DNA binding is modelled similarly in multiple tools, the major difference is present in the C-terminal domain. AlphaFold2 [17] (and OmegaFold [18]) predicts here one long helix (residues 124-155) and two short helices (residues 93-99 and 102-113). In contrast, ESM [19] (and RGN2 [20], and Phyre2 [21]) merge those shorthelices into single, long helix (93-113). The letter hypothesis is supported additionally by secondary structure predictions and oligomerisation tendency (only two long helices in C-terminal domain allow to predict/form well-defined oligomers as presented in Figure 7B).

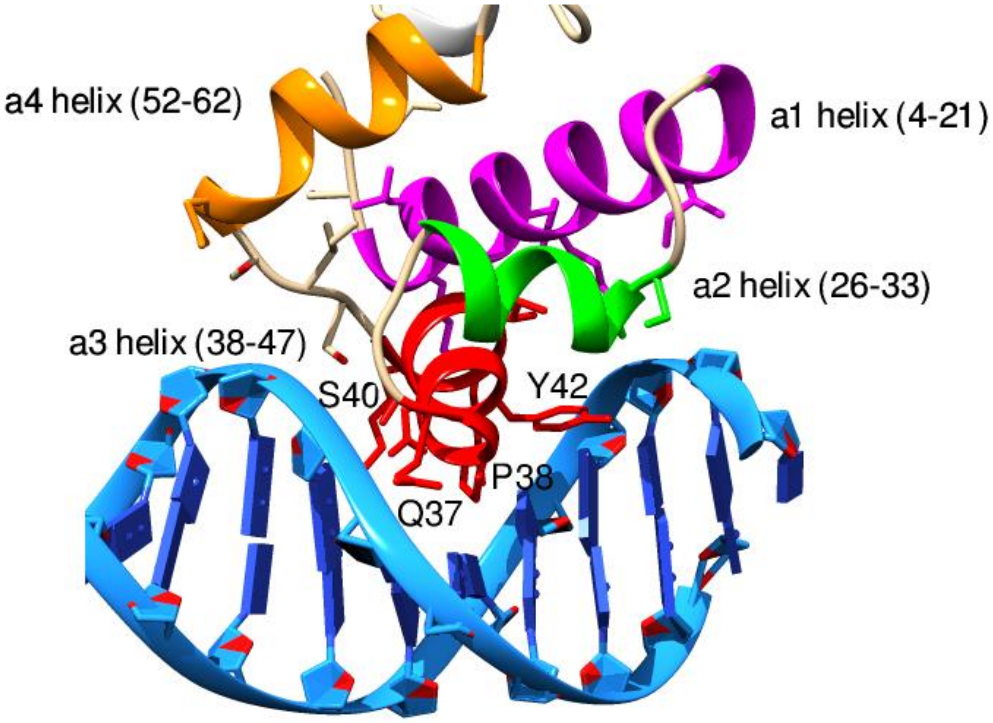

Figure S8. Binding of the N-terminal domain (NTD) of RacR to DNA. Bioinformatics modeling of the NTD's 3D structure reveals the presence of four helices with a common fold observed in several transcription factors, including the C regulator. Critical interactions are primarily dictated by the α3 helix via major grove of DNA (residues 38-47, with key interaction residues: Q37, P38, S40, Y42), with some contribution from the α2 helix. Meanwhile, the α1 and α4 helices serve as the structural scaffold for the domain. Model has been generated by ESM program [19] and aligned to PDB:1LLI template to infer DNA binding.

Differential expression analysis of transcriptomes with the C gene reveal changes in the expression profile of genes identified by Tn*5* mutagenesis

In our previous study, we performed transcriptome analysis for non-filamentous (p30) vs filamentous (p18) cells when transcriptional cross-talk was induced by the C protein (3). Main changes were identified within the Rac prophage. Now, having a large data set available from the whole transcriptome analysis, we aimed to check whether the expression pattern of genes which are now disrupted by the Tn*5* cassette was different between filamentous and non-filamentous when they were present in the genome. The analysis showed that among 41 considered genes 10 had significantly (more than 2 fold) changed expression: 8 were down-regulated and two slightly up-regulated (Figure S2). The highest change was detected for *nlpA* coding for lipoprotein 28. Only two genes were slightly up-regulated among them *wcaD* of colonic acid polymerase which, together with *rcsA*, are engaged in exopolysaccharide (EPS) pathway.

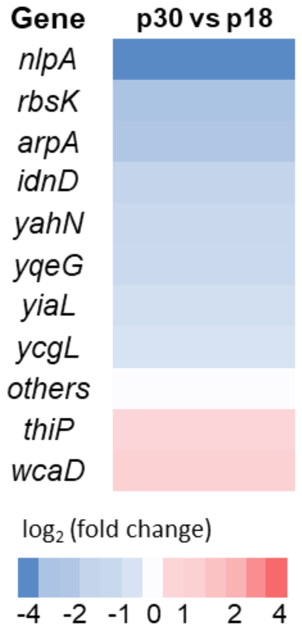

Figure S9. Changes in the expression profile of genes identified by Tn*5* mutagenesis in non-filamentous (p30) vs filamentous (p18) cells induced by cross-talk. Note that expression analysis refers to genes now identified by Tn5 mutagenesis but the results come from previous transcription analysis when the cross-talk was induced in the same manner (C protein) but in wild type MG1655 strain (not Tn5 mutant, where obviously these genes are interrupted).

Other regulators

Gene	Fold change
cyaA	nc
crp	4,43 ↓
rpoS	nc
rpoE	2,18 ↓
rcsA	nc
rcsB	3,71 ↓
hns	4,66 ↓
rfaH	nc
flhD	8,80 ↓
flhC	8,00 ↓

Lipopolysaccharide synthesis

Gene	Fold change
waaB	3,71 ↓
waaG	2,17 ↓
waaL	2,79 ↓
waaO	2,53 ↓
waaP	2,40 ↓
waaR	3,04 ↓
waaS	2,39 ↓
waaU	3,35 ↓
waaY	3,19 ↓
waaZ	2,42 ↓
wbbI	3,21 ↓
wbbJ	4,63 ↓
wbbK	4,91 ↓
gmhB	2,63 ↓
glf	3,39 ↓
lpxP	3,18 ↓

Colanic acid synthesis

Gene	Fold change
wza	5,48 ↑
wzb	nc
wcaC	2,55 ↑
wzy	nc
wcaE	2,40 ↑
wcaF	2,34 ↑
gmd	nc
wcaG	3,51 ↑
wcaH	nc
wcaI	2,12 ↑
manC	nc
wcaJ	2,10 ↑
wcaK	nc
wcaL	nc

Z-ring protein genes

Gene	Fold change
ftsZ	nc
ftsA	nc
ftsK	nc
ftsQ	nc
ftsL	nc
ftsB	nc
ftsW	nc
ftsI	2,85 ↓
ftsN	nc
ftsE	3,62 ↑
ftsX	2,57 ↑
ftsP	2,68 ↑
amiC	nc

Z-ring associated proteins

Gene	Fold change
zapA	nc
zapB	2,74 ↓
zapC	2,23 ↓
zapD	2,89 ↑
zapE	nc
minD	4,37 ↓
minE	4,52 ↓
matP	7,31 ↓

Transcription factors of cell division

Gene	Fold change
mraZ	nc
sdiA	2,32 ↑
dicA	2,53 ↓

Inhibitors of FtsZ/cell division

Gene	Fold change
kilR	200,06 ↑
sulA	nc

Figure S10. Selected transcriptomic data for *E. coli* MG1655 cells expressing C gene vs. C-absent (4). The results have been deposited in the NCBI GEO (accession number GSE126248).

Reference list:

1. Blattner, F.R., Plunkett, G., Bloch, C.A., Perna, N.T., Burland, V., Riley, M., Collado-Vides, J., Glasner, J.D., Rode, C.K., Mayhew, G.F. *et al.* (1997) The complete genome sequence of *Escherichia coli* K-12. *Science*, **277**, 1453-1462.
2. Baba, T., Ara, T., Hasegawa, M., Takai, Y., Okumura, Y., Baba, M., Datsenko, K.A., Tomita, M., Wanner, B.L. and Mori, H. (2006) Construction of Escherichia coli K-12 in-frame, single-gene knockout mutants: the Keio collection. *Mol Syst Biol*, **2**, 2006.0008.
3. Wang, X., Kim, Y., Ma, Q., Hong, S.H., Pokusaeva, K., Sturino, J.M. and Wood, T.K. (2010) Cryptic prophages help bacteria cope with adverse environments. *Nat Commun*, **1**, 147.
4. Negri, A., Jąkalski, M., Szczuka, A., Pryszcz, L.P. and Mruk, I. (2019) Transcriptome analyses of cells carrying the Type II Csp231I restriction-modification system reveal cross-talk between two unrelated transcription factors: C protein and the Rac prophage repressor. *Nucleic Acids Res*, **47**, 9542-9556.
5. Metcalf, W.W., Jiang, W., Daniels, L.L., Kim, S.K., Haldimann, A. and Wanner, B.L. (1996) Conditionally replicative and conjugative plasmids carrying lacZ alpha for cloning, mutagenesis, and allele replacement in bacteria. *Plasmid*, **35**, 1-13.
6. Casadaban, M.J. and Cohen, S.N. (1980) Analysis of gene control signals by DNA fusion and cloning in *Escherichia coli*. *J Mol Biol*, **138**, 179-207.
7. Miller, V.L. and Mekalanos, J.J. (1988) A novel suicide vector and its use in construction of insertion mutations: osmoregulation of outer membrane proteins and virulence determinants in Vibrio cholerae requires toxR. *J Bacteriol*, **170**, 2575-2583.
8. Zhou, L., Yu, H., Wang, K., Chen, T., Ma, Y., Huang, Y., Li, J., Liu, L., Li, Y., Kong, Z. *et al.* (2020) Genome re-sequencing and reannotation of the Escherichia coli ER2566 strain and transcriptome sequencing under overexpression conditions. *BMC Genomics*, **21**, 407.
9. Fomenkov, A., Sun, Z., Dila, D.K., Anton, B.P., Roberts, R.J. and Raleigh, E.A. (2017) EcoBLMcrX, a classical modification-dependent restriction enzyme in Escherichia coli B: Characterization in vivo and in vitro with a new approach to cleavage site determination. *PLoS One*, **12**, e0179853.
10. Larsen, R.A., Wilson, M.M., Guss, A.M. and Metcalf, W.W. (2002) Genetic analysis of pigment biosynthesis in Xanthobacter autotrophicus Py2 using a new, highly efficient transposon mutagenesis system that is functional in a wide variety of bacteria. *Arch Microbiol*, **178**, 193-201.
11. Guzman, L.M., Belin, D., Carson, M.J. and Beckwith, J. (1995) Tight regulation, modulation, and high-level expression by vectors containing the arabinose PBAD promoter. *J Bacteriol*, **177**, 4121-4130.
12. Rezulak, M., Borsuk, I. and Mruk, I. (2016) Natural C-independent expression of restriction endonuclease in a C protein-associated restriction-modification system. *Nucleic Acids Research*, **44**, 2646-2660.
13. Dylewski, M., Ćwiklińska, M. and Potrykus, K. (2018) A search for the in trans role of GraL, an Escherichia coli small RNA. *Acta Biochim Pol*, **65**, 141-149.
14. Potrykus, K., Vinella, D., Murphy, H., Szalewska-Palasz, A., D'Ari, R. and Cashel, M. (2006) Antagonistic regulation of Escherichia coli ribosomal RNA rrnB P1 promoter activity by GreA and DksA. *J Biol Chem*, **281**, 15238-15248.
15. Wisniewska, A., Wons, E., Potrykus, K., Hinrichs, R., Gucwa, K., Graumann, P.L. and Mruk, I. (2022) Molecular basis for lethal cross-talk between two unrelated bacterial transcription factors - the regulatory protein of a restriction-modification

system and the repressor of a defective prophage. *Nucleic Acids Res*, **50**, 10964-10980.

16. Mercer, K.L. and Weiss, D.S. (2002) The Escherichia coli cell division protein FtsW is required to recruit its cognate transpeptidase, FtsI (PBP3), to the division site. *J Bacteriol*, **184**, 904-912.
17. Jumper, J., Evans, R., Pritzel, A., Green, T., Figurnov, M., Ronneberger, O., Tunyasuvunakool, K., Bates, R., Žídek, A., Potapenko, A. *et al.* (2021) Highly accurate protein structure prediction with AlphaFold. *Nature*, **596**, 583-589.
18. Wu R., Ding F., Wang R., Shen R., Zhang X., Luo S., Su C., Wu Z., Xie Q., Berger B. *et al.* (2022). High-resolution *de novo* structure prediction from primary sequence. bioRxiv 2022.07.21.500999; doi: https://doi.org/10.1101/2022.07.21.500999.
19. Lin, Z., Akin, H., Rao, R., Hie, B., Zhu, Z., Lu, W., Smetanin, N., Verkuil, R., Kabeli, O., Shmueli, Y. *et al.* (2023) Evolutionary-scale prediction of atomic-level protein structure with a language model. *Science*, **379**, 1123-1130.
20. Chowdhury, R., Bouatta, N., Biswas, S., Floristean, C., Kharkar, A., Roy, K., Rochereau, C., Ahdritz, G., Zhang, J., Church, G.M. *et al.* (2022) Single-sequence protein structure prediction using a language model and deep learning. *Nat Biotechnol*, **40**, 1617-1623.
21. Kelley, L.A., Mezulis, S., Yates, C.M., Wass, M.N. and Sternberg, M.J. (2015) The Phyre2 web portal for protein modeling, prediction and analysis. *Nat Protoc*, **10**, 845-858.
